# Single-molecule genome-wide mutation profiles of cell-free DNA for non-invasive detection of cancer

**DOI:** 10.1038/s41588-023-01446-3

**Published:** 2023-07-27

**Authors:** Daniel C. Bruhm, Dimitrios Mathios, Zachariah H. Foda, Akshaya V. Annapragada, Jamie E. Medina, Vilmos Adleff, Elaine Jiayuee Chiao, Leonardo Ferreira, Stephen Cristiano, James R. White, Sarah A. Mazzilli, Ehab Billatos, Avrum Spira, Ali H. Zaidi, Jeffrey Mueller, Amy K. Kim, Valsamo Anagnostou, Jillian Phallen, Robert B. Scharpf, Victor E. Velculescu

**Affiliations:** 1 The Sidney Kimmel Comprehensive Cancer Center, Johns Hopkins University School of Medicine, Baltimore, MD USA; 2 Division of Computational Biomedicine, Department of Medicine, Boston University, Boston, MA USA; 3 Section of Pulmonary and Critical Care Medicine, Department of Medicine, Boston University, Boston, MA USA; 4 Allegheny Health Network Cancer Institute, Allegheny Health Network, Pittsburgh, PA USA

**Keywords:** Medical research, Cancer, Sequencing

## Abstract

Somatic mutations are a hallmark of tumorigenesis and may be useful for non-invasive diagnosis of cancer. We analyzed whole-genome sequencing data from 2,511 individuals in the Pan-Cancer Analysis of Whole Genomes (PCAWG) study as well as 489 individuals from four prospective cohorts and found distinct regional mutation type-specific frequencies in tissue and cell-free DNA from patients with cancer that were associated with replication timing and other chromatin features. A machine-learning model using genome-wide mutational profiles combined with other features and followed by CT imaging detected >90% of patients with lung cancer, including those with stage I and II disease. The fixed model was validated in an independent cohort, detected patients with cancer earlier than standard approaches and could be used to monitor response to therapy. This approach lays the groundwork for non-invasive cancer detection using genome-wide mutation features that may facilitate cancer screening and monitoring.

## Abstract (web summary)

Somatic mutations are identified from circulating cell-free DNA using a single-molecule-based lowpass whole-genome sequencing method. The regional distribution of mutations can identify a tumor-specific mutational profile in patients with cancer and can be used to monitor patients through treatment.

## Main

Most human mortality associated with cancer is a consequence of diagnosis at late stages, when therapies are less effective^1^. Early detection of cancer has demonstrated clinical benefits in multiple cancer types, but the implementation of screening approaches remains challenging^2^. For example, screening for lung cancer using low-dose computed tomography (LDCT) is recommended in the United States for adults aged 50–80 years who have smoked at least 20 pack years and currently smoke or have quit smoking within the last 15 years^3^. Although screening with LDCT has been shown to reduce mortality^4,5^, adherence to this test is low (<6%) among high-risk individuals^6^, in part owing to the potential harm caused by its low specificity, radiation exposure and unnecessary diagnostic procedures as a result of overdiagnosis. For other cancers, although early detection could improve patient outcomes, no effective screening modalities are available^7^. Liquid biopsies may overcome these challenges and provide an attractive approach for the non-invasive detection of lung cancer and other malignancies.

Sequence alterations are abundant in cancer genomes but the proportion of fragments in cell-free DNA (cfDNA) that harbor tumor-specific (somatic) mutations is often low^8,9^, making it difficult to detect bona fide variants amidst background noise from sequence changes introduced in library construction and sequencing. Extensive efforts have been made to detect low-frequency mutations in cfDNA. However, these methods typically rely on deep sequencing and have been restricted to examining specific genes comprising a small subset of the genome^10–12^. Owing to the low number of tumor genome equivalents in cfDNA, such approaches have limited efficacy for detecting cancer, especially at early stages^13–15^. Additionally, cfDNA sequence alterations may arise from white blood cells, confounding cancer detection^8,16,17^. Recent analyses have shown that genome-wide fragmentation and methylation analyses could be used for non-invasive early cancer detection^13,14,18,19^.

Here, we considered whether identifying somatic mutations genome-wide could enable the detection of an increased number of circulating tumor DNA (ctDNA) alterations and increase the detection of early stage disease. Tumor genomes contain thousands of somatic changes^20,21^, and knowledge of such alterations from tumor tissue has guided ctDNA analyses during therapy^22,23^. In principle, if mutations could be identified in cfDNA without knowledge of alterations in the tumor, then they could be useful for early cancer detection. However, this would require efficient detection of somatic changes in cfDNA, including those related to mutational signatures^24^, and the ability to effectively distinguish these from non-tumor-derived alterations.

**Fig. 1:**
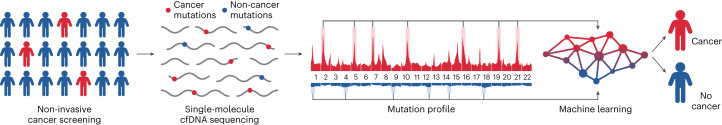
Schematic of overall approach for cancer detection using single-molecule cfDNA sequencing. Blood is collected from a population of individuals, some of whom have cancer. Then, cfDNA is extracted from plasma and subjected to single-molecule sequencing using massively parallel sequencing approaches. Sequence alterations are used to obtain genome‐wide mutation profiles, and regional differences in cancer and non-cancer mutation frequencies are identified using machine learning to distinguish individuals with and without cancer.

To address these challenges, we developed an approach, called GEnome-wide Mutational Incidence for Non-Invasive detection of cancer (GEMINI), that can identify a much larger number of somatic alterations in cfDNA (Fig. 1). We applied this method to tissue and cfDNA samples from multiple patient cohorts (Supplementary Fig. 1). The method involves sequencing individual cfDNA molecules to estimate the mutation frequency and type of alteration genome-wide, using non-overlapping bins ranging in size from thousands to millions of bases. For each individual, the mutation type and frequency in genomic regions more commonly altered in cancer is compared to the profile from regions more frequently mutated in normal cfDNA to determine multiregional differences in mutation profiles. In this way, GEMINI enriches probable somatic mutations while accounting for individual variability in overall background changes.

## Results

### Genome-wide somatic mutation analyses of cancer tissues

**Fig. 2:**
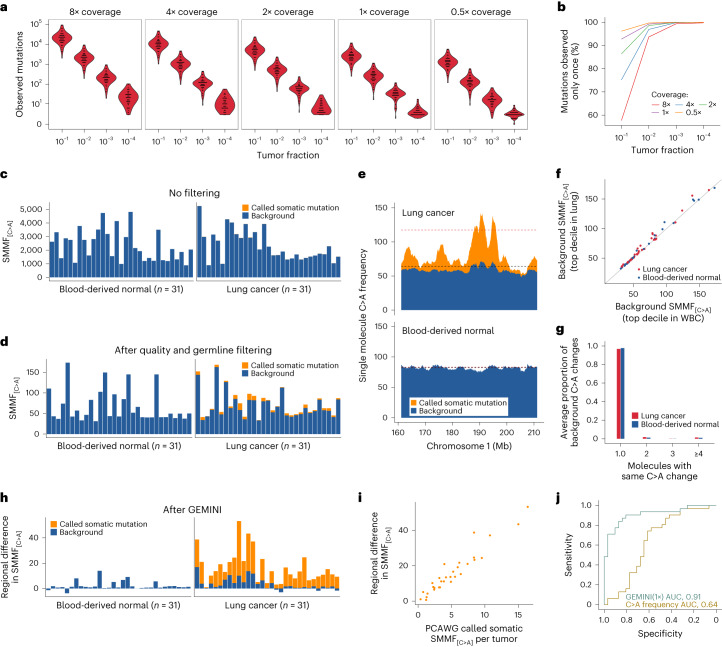
Single-molecule mutation analyses of PCAWG lung cancers and normal samples. **a**, Number of mutations detected in lung cancer samples from individuals who smoke, across sequencing coverage amounts and tumor fractions. **b**, Fraction of lung cancer mutations observed in single DNA molecules at the different coverage and tumor fractions indicated. **c**,**d**, Single-molecule mutation frequency (SMMF) for somatic and background C>A changes in lung cancer and blood-derived matched normal samples without quality or germline filters (**c**) or with these filters including filtering of 8-oxo-dG-related sequence changes (**d**). **e**, Frequency of single-molecule somatic and background C>A changes computed in a sliding 2.5 Mb window with a step size of 100 kb across a 50 Mb region of chromosome 1 in lung cancer and blood-derived normal samples from individual DO25320. Red and black dashed lines represent mutation frequencies of the top decile of bins most enriched in C>A changes in lung cancers and matched blood-derived normal samples. **f**, Background C>A frequency of the top decile of bins most enriched in C>A changes in lung cancer and matched white blood cell (WBC) samples obtained after removal of known somatic mutations. For each sample, background C>A frequencies are similar between these regions as can be seen with the solid identity line. **g**, Number of molecules with each background C>A change in lung cancer and blood-derived normal samples. Most background changes are observed only once. **h**, Regional C>A frequencies in normal or tumor samples after subtraction of the C>A frequency in the top decile of bins enriched in normal samples from the top decile of bins enriched in mutations in tumor samples. **i**, Regional differences in single-molecule C>A frequencies were positively correlated with the frequency of high-confidence somatic C>A mutations reported in these samples by the PCAWG Consortium (Spearman’s rho, 0.96; *P* < 0.0001, two-sided). **j**, Receiver operator characteristic curve for distinguishing lung cancer from normal samples using GEMINI with the testing set down-sampled to 1× coverage compared to using overall single-molecule C>A frequencies after quality and germline filtering. The GEMINI approach without filtering 8-oxo-dG-related changes results in an AUC of 0.47, highlighting the importance of removing these artefacts.

**Extended Data Fig. 1:**
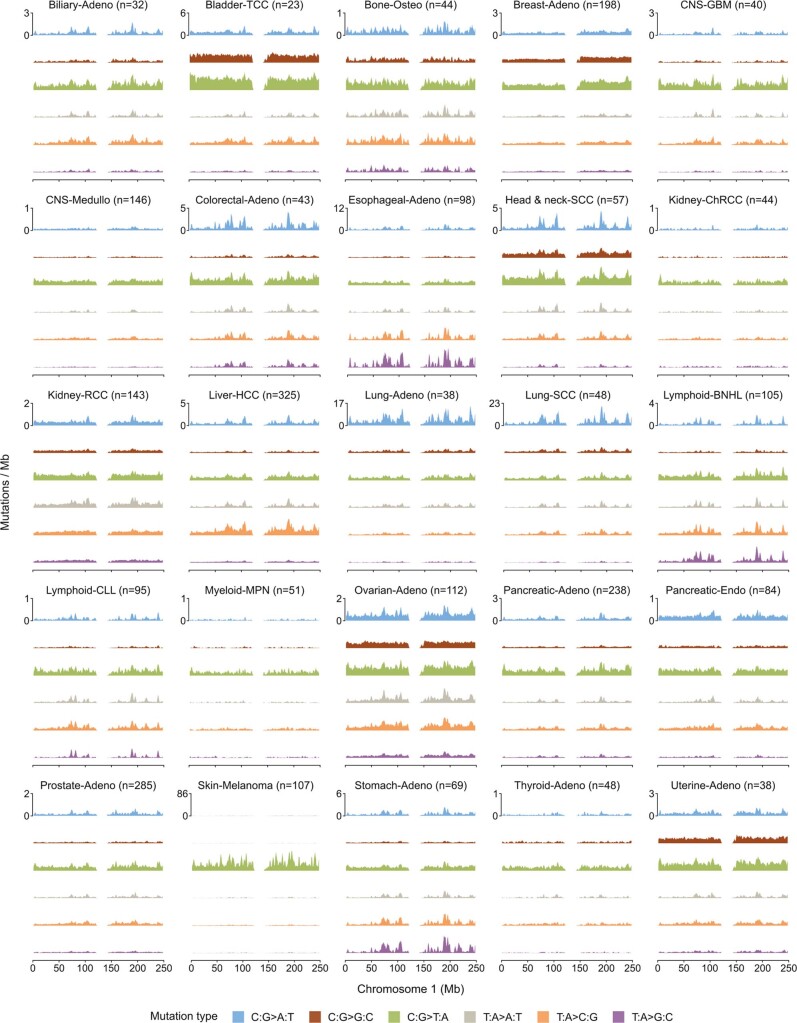
Genomic mutation profiles in common cancers. Average somatic mutation frequencies computed in sliding 2.5 megabase (Mb) windows with a step size of 100 kb across chromosome 1 obtained from an analysis of 2,511 PCAWG samples across 25 common cancer types. Adeno, adenocarcinoma; TCC, transitional cell carcinoma; Osteo, osteosarcoma; CNS, central nervous system; GBM, glioblastoma multiforme; Medullo, medulloblastoma; SCC, squamous cell carcinoma; ChRCC, chromophobe renal cell carcinoma; RCC, renal cell carcinoma; HCC, hepatocellular carcinoma; BNHL, B cell non-Hodgkin lymphoma; CLL, chronic lymphoid leukemia; MPN, myeloproliferative neoplasm; Endo, endocrine.

To develop this method, we examined whole-genome sequences of cancers from 2,511 individuals across 25 different cancers from the PCAWG study^25,26^, identifying distinct mutation frequencies across the genome in different tumor types (Extended Data Fig. 1). For example, analysis of lung tumor and matched normal tissue genomes from 65 individuals with smoking exposure revealed that the cancers had an average of 52,209 (range 6,031 to 193,539) bona fide somatic mutations per genome (Supplementary Table 1). In silico dilution and downsampling experiments revealed that these patients would theoretically have a subset of detectable mutations at tumor fractions as low as 1:10,000 using 1× coverage whole-genome sequencing (WGS) (Fig. 2a and Supplementary Fig. 2).

**Extended Data Fig. 2:**
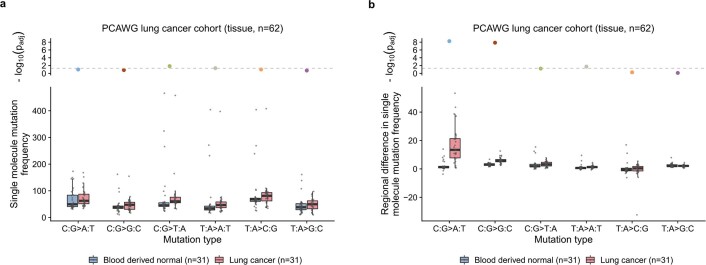
Analyses of single molecule sequence changes in PCAWG lung cancer and normal samples. **a**, Single molecule mutation frequencies in Pan-Cancer Analysis of Whole Genomes (PCAWG) lung cancers (n = 31) and blood derived matched normal samples (n = 31). Adjusted p-values (p_adj_) were generated using the two-sided Wilcoxon rank sum test and were corrected for multiple comparisons using the Benjamini-Hochberg method. The horizontal dashed line indicates a p-value of 0.05. **b**, Cross-validated regional differences in single molecule mutation frequencies in PCAWG lung cancers (n = 31) and blood derived matched normal samples (n = 31), median-centered within each mutation type. Adjusted p-values were generated using the two-sided Wilcoxon rank sum test and were corrected for multiple comparisons using the Benjamini-Hochberg method. The horizontal dashed line indicates a p-value of 0.05. All boxplots represent the interquartile range with whiskers drawn to the highest value within the upper and lower fences (upper fence = 0.75 quantile + 1.5 × interquartile range; lower fence = 0.25 quantile – 1.5 × interquartile range). The solid middle line in the boxplot corresponds to the median value.

As the majority of mutations detected at low coverage would be expected to be observed only once (Fig. 2b), we developed rigorous methods to examine the frequency of single-molecule somatic mutations in a mixture of germline changes, white blood cell alterations and experimental and sequencing artefacts (all considered background changes). We scanned each molecule for single-nucleotide changes and, after removing common germline variants and unevaluable regions, computed the frequency of putative mutations in high-quality reads, defined as the number of variants per million evaluated positions across all DNA molecules sequenced (Methods). As specific transversions probably related to the accumulation of 7,8-dihydro-8-oxoguanine (8-oxo-dG)^27^ were more abundant than expected from analyses of similar transversions at sites of known polymorphisms, we filtered these changes when they occurred in certain read combinations (Supplementary Fig. 3 and Methods). We examined these changes in PCAWG lung tumors with matched normal blood cells (*n* = 31), as blood cells represent the largest source of cfDNA in individuals that do not have cancer^28^. We focused our analyses on the remaining C:G>A:T mutations (hereafter referred to as C>A), given their high abundance in tumors from current and former smokers^29^. Given the high and variable overall frequency of background changes, C>A frequencies were similar in the tumors and normal samples (Fig. 2c), and were only slightly higher even after the filtering steps above and the removal of germline variants, in which only a small fraction of the tumor alterations were somatic in origin (average, 7.5%; range, 0.8–22%) (Fig. 2d, Extended Data Fig. 2a and Supplementary Fig. 4).

**Extended Data Fig. 3:**
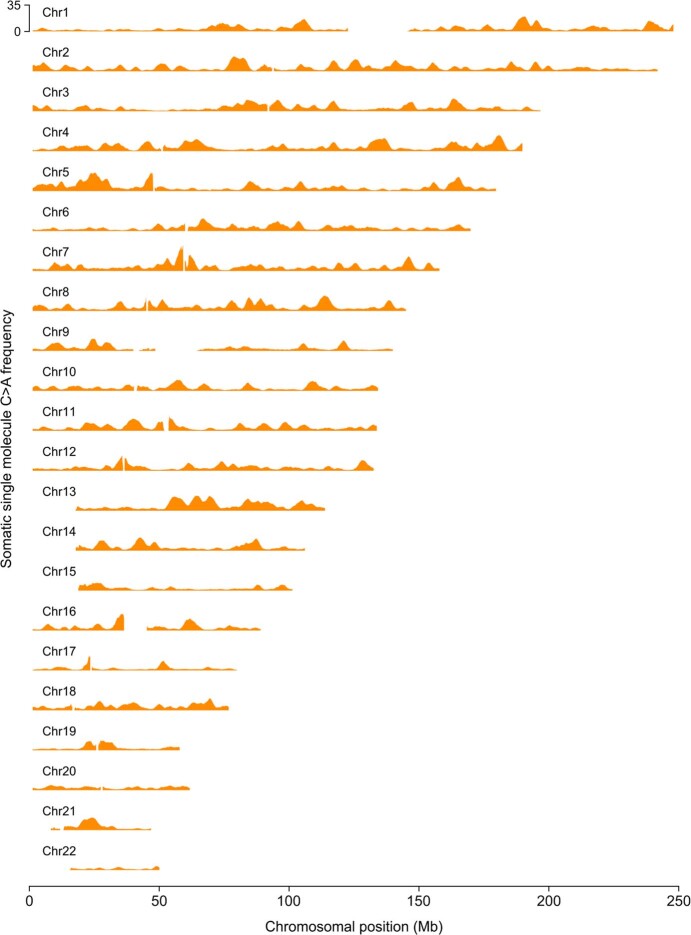
Genome‐wide somatic single molecule C > A mutation profiles in lung cancers. Single molecule C > A somatic mutation frequencies computed in sliding 2.5 megabase (Mb) windows with a step size of 100 kb across the autosomes obtained from an aggregated analysis of the 31 PCAWG lung cancer samples showed widespread differences in mutation frequencies depending on genomic location. Chr, Chromosome.

**Extended Data Fig. 4:**
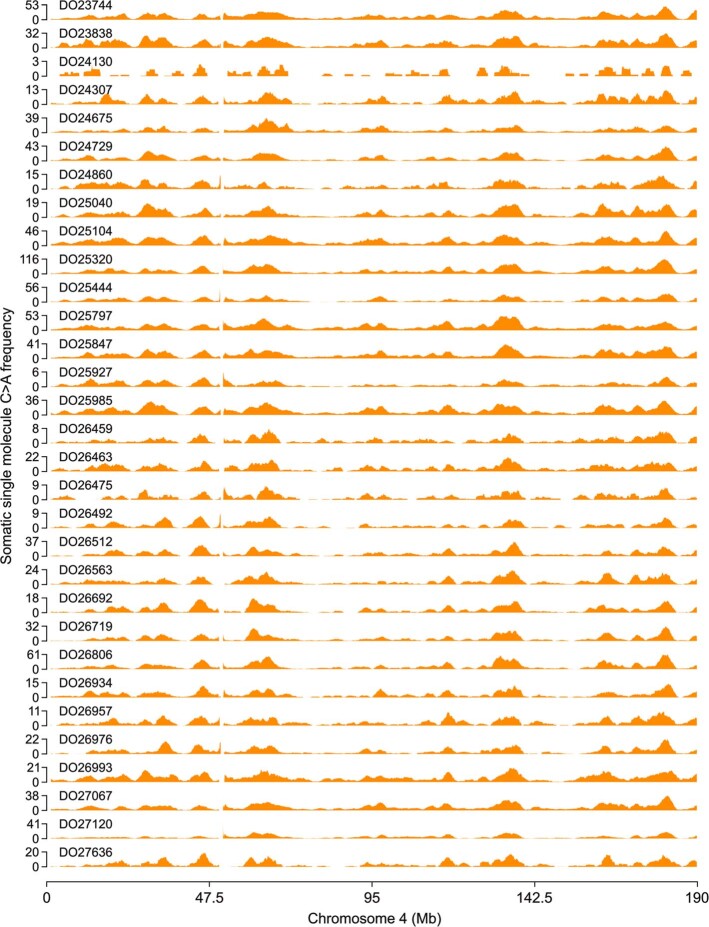
Somatic single molecule C > A mutation profiles across chromosome 4 in PCAWG lung cancers. Single molecule C > A somatic mutation frequencies computed in a sliding 2.5 megabase (Mb) window with a step size of 100 kb across chromosome 4 from PCAWG lung cancer samples (n = 31) revealed similar mutation profiles among different lung cancers. Patient IDs (for example DO23744) are indicated for each sample.

We investigated the high number and variability of total background changes among samples and found that these were largely related to sequencing lane-specific and run-specific artefacts (Supplementary Fig. 5). We reasoned that controlling for overall background rates in a sample-specific manner could improve the detection of tumor-derived changes. Previous analyses have shown that mutation rates differ across cancer genomes; regions associated with euchromatin, including expressed genes and early replicating regions, have a lower mutation rate than heterochromatin regions representing unexpressed genes and late-replicating regions^30,31^. To examine the variation in mutation frequency across the genome, we analyzed the 31 PCAWG paired samples by binning the sequence data containing 3,076,901 mutations into 1,144 non-overlapping 2.5 megabase (Mb) bins and found regions throughout the genome with increased mutation frequencies shared by many tumors (Extended Data Figs. 3 and 4).

**Extended Data Fig. 5:**
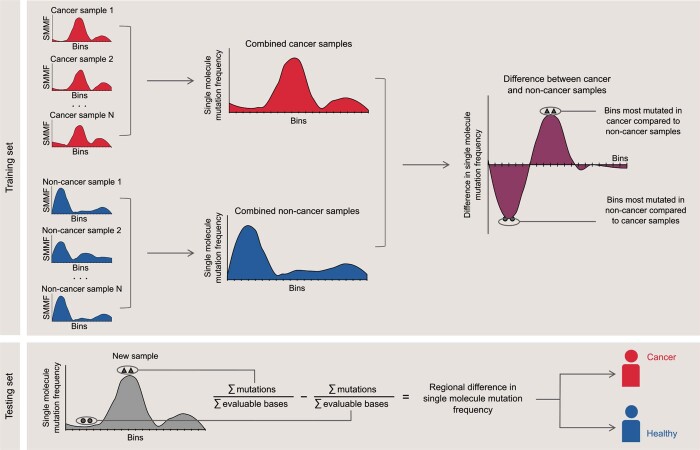
Schematic of GEMINI regional mutation frequency analysis. The genome is divided into 1,144 non-overlapping 2.5 Mb bins (20 bins are depicted here) and the single molecule mutation frequency (SMMF) is computed in each bin as the number of sequence changes per million evaluable bases, defined as the number of positions in fragments in which each sequence change could be detected after quality and germline filtering. Samples in the training set are used to identify the bins that are most differentially mutated between cancer and non-cancer samples. In the training set, sequence data from all cancer samples and all non-cancer samples are combined, and the cancer and non-cancer single molecule mutation frequencies are computed in each bin. Next, the difference in single molecule mutation frequency is computed between cancer and non-cancer samples in each bin, and the 10% of bins most mutated in cancer samples relative to non-cancer samples, as well as the 10% of bins most mutated in non-cancer samples relative to cancer samples, are identified (indicated by triangles and circles respectively). In the testing set, the difference in single molecule mutation frequency is computed between these two sets of bins in a new sample not included in the training set, generating a regional difference in mutation frequency that can be used to classify the sample into being derived from a healthy individual or an individual with cancer. By taking the difference in single molecule mutation frequency between two sets of regions in the genome within an individual sample, this approach controls for the overall number of sequence changes in that sample that may result from technical variability in sequencing runs.

To evaluate GEMINI for the detection of tumor-derived DNA, we identified genomic regions with the highest C>A changes in a training set of cancers and controls and then computed the average C>A difference at these regions for patients not represented in the training set (Extended Data Fig. 5 and Methods). We identified regions enriched for C>A changes in the 31 PCAWG cancers but not in normal samples (Fig. 2e) and found that background changes were highly correlated in cancer and control regions for each patient sample (Pearson’s correlation coefficient, 0.99; *P* < 0.0001) (Fig. 2f), suggesting that subtraction of alteration frequencies between cancer and control regions within a given patient sample would be useful for removing background mutations. By contrast, subtraction of specific mutations observed in the matched normal sample from the single-molecule sequencing data was ineffective at removing background changes (Supplementary Fig. 6) because such alterations typically occurred de novo and were seen once (Fig. 2g). After background subtraction, the remaining regional mutation frequencies were appreciably higher in tumors compared to normal samples (median of 13.4 compared to 1.3, respectively; Wilcoxon rank sum test, *P* < 0.0001). A high fraction of changes resulted from somatic mutations (average, 80%; range, 31–100%) (Fig. 2h) and were highly correlated with the frequency of high-confidence somatic C>A changes reported in these samples by the PCAWG consortium (Pearson’s correlation coefficient, 0.96; *P* < 0.0001) (Fig. 2i). Using C>A regional frequencies, GEMINI distinguished PCAWG cancer from non-cancer samples with high accuracy (area under the curve (AUC), 0.91; 95% confidence interval (CI), 0.84–0.99) compared to mutation frequencies alone (AUC, 0.64; 95% CI, 0.50–0.79) using low-coverage WGS (Fig. 2j and Extended Data Fig. 2b). The overall approach for filtering background changes resulted in a 1,903-fold enrichment in somatic mutations in these samples (Supplementary Table 2).

### Detection of cancer type-specific mutation profiles in cfDNA

We next evaluated the ability of GEMINI to detect sequence alterations in cfDNA from individuals from a prospective lung cancer diagnostic cohort (LUCAS)^18^. We analyzed low-coverage plasma WGS data (~2× coverage) from the 365 individuals examined in this trial, the majority of whom were at high risk for lung cancer (aged 50–80 years with a ≥20 pack-year smoking history; Supplementary Table 3). Given the short length of cfDNA fragments^13^, we restricted our analyses to regions with identical sequence calls in overlapping reads in the paired-end library (Supplementary Table 2). This would theoretically reduce the sequencing error rate and confer the benefits of a higher degree of overlap for shorter tumor-derived cfDNA sequences^32^, thereby potentially enriching ctDNA alterations.

**Fig. 3:**
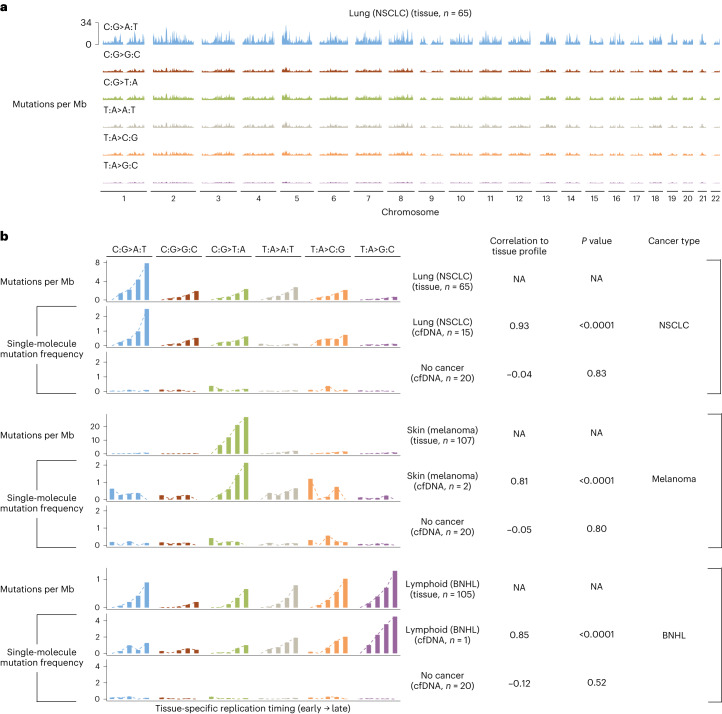
Genome-wide mutation profiles of tissue and plasma samples are associated with replication timing. **a**, Somatic mutation frequencies in PCAWG lung cancers of individuals who smoke (*n* = 65) were computed in sliding 2.5 Mb windows with a step size of 100 kb across the genome and are represented as the average across individuals. **b**, Association of mutation frequencies across tissue-specific replication timing strata in PCAWG tissue samples and cfDNA from patients in the LUCAS cohort with NSCLC, melanoma, B cell non-Hodgkin lymphoma (BNHL) or no cancer. Replication timing was obtained as the wavelet‐smoothed transform of the six fraction profile, representing different time points during replication in 1 kb bins from IMR90, NHEK and GM12878 cell lines for analyses of NSCLC, melanoma and BNHL, respectively. The weighted average of the replication timing values was computed in 2.5 Mb bins, followed by grouping of bins into five equal bin sets containing bins with the earliest to latest replication timing. In each bin set, we computed the mutation frequency in tissue at different replication strata using the number of somatic mutations reported by the PCAWG Consortium per Mb of genome and compared this to the single-molecule mutation frequency in plasma using a two-sided Pearson’s correlation. To control for potential systematic variability in measured genome-wide mutational frequencies, we subtracted from both cancer and non-cancer cfDNA samples the single-molecule mutation frequency in each bin set in a separate panel of 20 non-cancer cfDNA samples. Mutation frequencies were then scaled within each sample and mutation type to have a minimum value of zero. NA, not applicable.

We found that genome regions with a high frequency of mutations were largely similar between tumor tissue and cfDNA from patients with lung cancer, melanoma and B cell non-Hodgkin lymphoma (Pearson’s correlation, >0.80; *P* < 0.0001 in all cases) and were located in genomic regions associated with tissue-specific late replication timing (Fig. 3). Different mutation types among the tumors analyzed contributed to the high mutation frequencies, including C>A changes in lung cancer, C>T in melanoma and T>G in lymphoma. We also found that tumor- and mutation type-specific regional mutation frequencies were related to gene expression^30^, genome compartmentalization as measured by eigenvector analyses of methylation^33^, as well histone 3 lysine 9 trimethylation (H3K9me3), a known mark of heterochromatin^34^, and were consistent between tumor and cfDNA analyses (Pearson’s correlation, >0.80; *P* < 0.0001 in all cases) (Supplementary Fig. 7). Individuals without cancer or mutation types or regions not enriched in cancer did not have or were weakly correlated with these characteristics (Fig. 3b and Supplementary Fig. 7). Overall, these results suggest that genome-wide mutation rate variability in cfDNA is related to chromatin organization and can be leveraged by GEMINI to detect tumor-derived sequence changes in the circulation.

### Non-invasive detection of lung cancer with GEMINI

**Extended Data Fig. 6:**
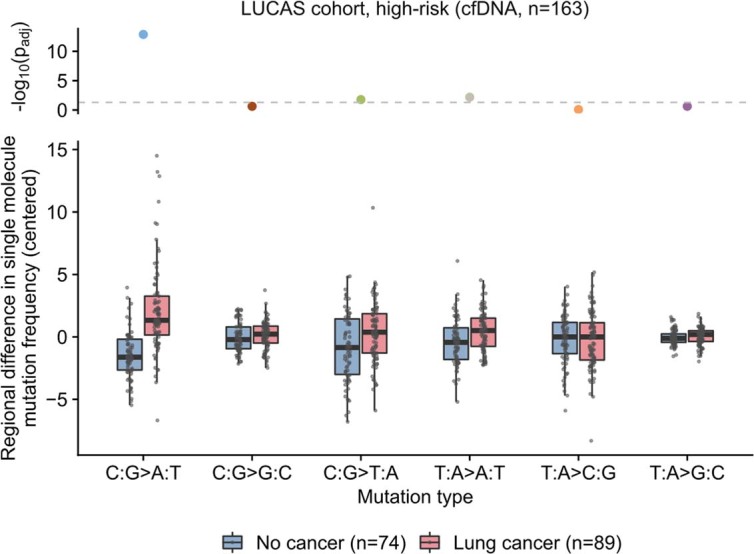
Regional differences in single molecule mutation frequencies in the high-risk LUCAS cohort. Cross-validated regional differences in single molecule mutation frequencies in cell-free DNA (cfDNA) in individuals with lung cancer (n = 89) and individuals without cancer (n = 74), median-centered within each mutation type. Regional C > A mutation frequencies were preferentially altered between lung cancer and non-cancer samples, but not when randomly permuting class labels (p = 0.36, Wilcoxon rank sum test, two-sided). Adjusted p-values (p_adj_) were generated using the two-sided Wilcoxon rank sum test and were corrected for multiple comparisons using the Benjamini-Hochberg method. The horizontal dashed line indicates a p-value of 0.05. All boxplots represent the interquartile range with whiskers drawn to the highest value within the upper and lower fences (upper fence = 0.75 quantile + 1.5 × interquartile range; lower fence = 0.25 quantile – 1.5 × interquartile range). The solid middle line in the boxplot corresponds to the median value.

Using GEMINI, we identified cross-validated regional differences in mutation frequencies for individuals in the LUCAS cohort. Similar to analyses in PCAWG lung cancers, regional C>A mutation frequencies were preferentially altered in samples from individuals with lung cancer compared to those without (Wilcox rank sum test, *P* < 0.0001) (Extended Data Fig. 6). Regional C>A mutation frequencies were not related to estimated levels of 8-oxo-dG changes (Spearman’s rho, −0.02; *P* = 0.80) and, unlike overall C>A frequencies, they were stable across sequencing lanes (Supplementary Fig. 8). The regions identified were largely consistent across cross-validation folds and comprised high-quality sequences with similar evaluable bases, copy number levels and mappability but were located at positions reflecting the epigenomic characteristics described above (Supplementary Fig. 9). We further compared the regional differences in C>A mutation frequencies to CC>AA doublet mutations because these are enriched in lung cancers of individuals who smoke^26^ and they have a very low likelihood of occurring by chance given the requirement of two identical changes occurring in adjacent positions (Supplementary Fig. 10). The frequency of high-quality CC>AA changes was highly correlated with the regional difference in C>A frequency in both tissue (Spearman’s rho, 0.62; *P* = 0.0002) and cfDNA samples (Spearman’s rho, 0.65; *P* < 0.0001) (Supplementary Fig. 10e,f), supporting the idea that GEMINI mutational frequencies reflect tumor-derived sequence changes in the circulation.

**Fig. 4:**
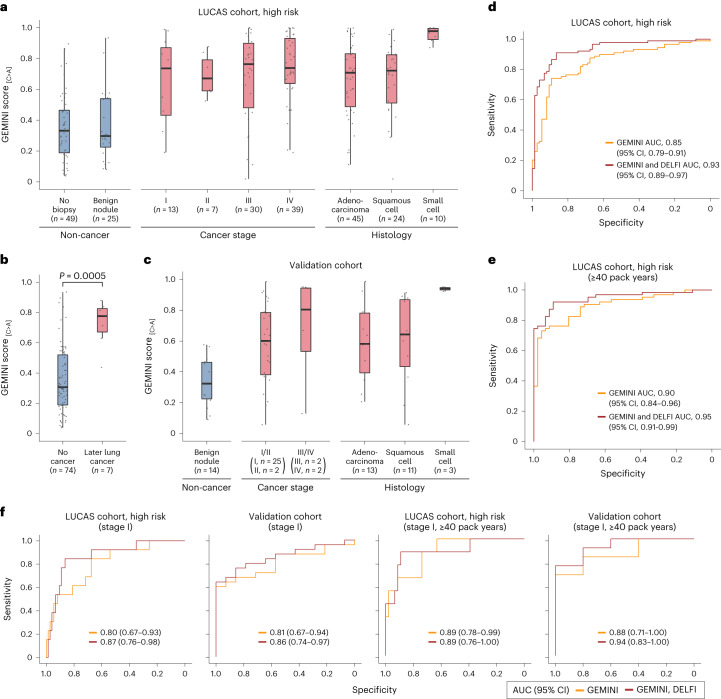
Detection of lung cancer using GEMINI and a combined GEMINI–DELFI approach. **a**, Cross-validated GEMINI scores in the LUCAS cohort of high‐risk individuals (aged 50–80 years with a ≥20 pack-year smoking history with or without lung cancer), with the number of individuals indicated at each stage or histology. **b**, GEMINI scores of high‐risk individuals without lung cancer as well as individuals without lung cancer as determined by imaging at baseline but who later developed lung cancer. The difference between groups was evaluated using a two-sided Wilcoxon rank sum test. **c**, The fixed GEMINI model from the LUCAS cohort was used to evaluate individuals in a validation cohort of current or former smokers aged 50–80 years with and without cancer. **d**, Receiver operator characteristic (ROC) curve for detection of lung cancer in high‐risk individuals in the LUCAS cohort (*n* = 89 with lung cancer, *n* = 74 without cancer). **e**, ROC curve for detection of lung cancer in a subset of high‐risk individuals in the LUCAS cohort with at least 40 pack years (*n* = 63 with lung cancer, *n* = 46 without cancer) shows that the performance of GEMINI is better with higher smoking history. **f**, ROC curve for detection of high‐risk individuals from the LUCAS cohort who were diagnosed with stage I lung cancer (*n* = 13 with lung cancer, *n* = 74 without cancer) (left panel), stage I lung cancer among individuals in the validation cohort (*n* = 25 with lung cancer, *n* = 14 without cancer) (middle-left panel), high‐risk individuals from the LUCAS cohort with a ≥40 pack-year smoking history who were diagnosed with stage I lung cancer (*n* = 9 with lung cancer, *n* = 46 without cancer) (middle-right panel) and stage I lung cancer among individuals with a ≥40 pack-year smoking history in the validation cohort (*n* = 13 with lung cancer, *n* = 5 without cancer) (right panel). All boxplots represent the interquartile range, with whiskers drawn to the highest value within the upper and lower fences (upper fence, 0.75 quantile + 1.5× interquartile range; lower fence, 0.25 quantile – 1.5× interquartile range). The solid middle line in the boxplot represents the median value.

**Extended Data Fig. 7:**
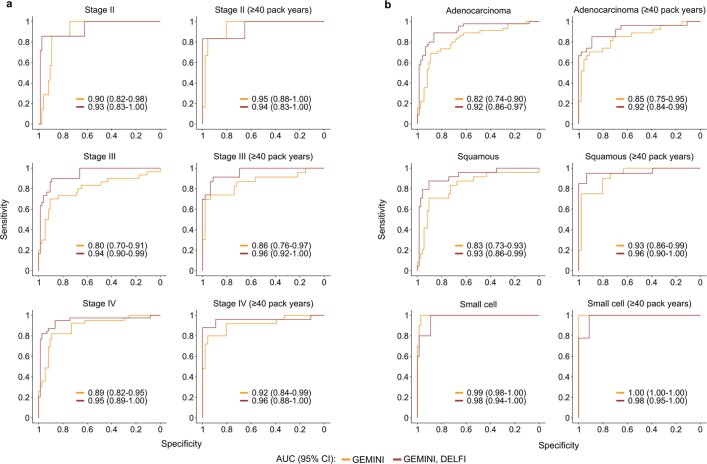
Performance of GEMINI or the combined GEMINI / DELFI approach for detection of lung cancer. **a**, ROC curves for detection of lung cancer in the high-risk LUCAS cohort using GEMINI or the combined GEMINI / DELFI approach in patients with stages II-IV disease and in the subset of these patients that smoked ≥40 pack years. **b**, ROC curves for detection of lung cancer in the high-risk LUCAS cohort using GEMINI or the combined GEMINI / DELFI approach in patients with adenocarcinoma, squamous cell carcinoma, or small cell lung cancer and in the subset of these patients that smoked ≥40 pack years. Performance for Stage I disease is shown in Fig. 4f. AUC, area under the curve; CI, confidence interval.

We calibrated the regional differences in C>A frequencies to GEMINI scores, reflecting an individual’s probability of having cancer (Methods). GEMINI scores were similar in individuals without cancer, with and without benign lesions (median GEMINI score, 0.30 versus 0.33; Wilcoxon rank sum test, *P* = 0.94) (Fig. 4a), and were not associated with demographic characteristics or the presence of acute or chronic inflammatory conditions (Supplementary Fig. 11). By contrast, patients with cancer had significantly higher median scores than individuals without cancer across all stages (stage I, 0.74; stage II, 0.67; stage III, 0.76; stage IV, 0.74) (Wilcoxon rank sum test, *P* < 0.001 for stages I, II, III and IV) (Fig. 4a) and histological subtypes (adenocarcinoma, 0.71; squamous cell carcinoma, 0.72; small cell lung cancer (SCLC), 0.98) (Wilcoxon rank sum test, *P* < 0.0001 for all subtypes) (Fig. 4a). GEMINI scores were generally related to ctDNA levels, increasing with estimated tumor fractions^35^ (Wilcoxon rank sum test, *P* < 0.0001) (Supplementary Fig. 12a). Higher GEMINI scores in patients with SCLC probably reflected the known higher ctDNA fractions in this tumor type^36^. A receiver operator characteristic curve representing the sensitivity and specificity of GEMINI to identify cancer in patients revealed an overall AUC of 0.85 (95% CI, 0.79–0.91) (Fig. 4d), with high performance across stages and subtypes (Fig. 4f and Extended Data Fig. 7a,b).

The fixed GEMINI model was used to evaluate samples from seven patients who did not have any detectable tumors at the time of blood collection, using standard imaging and diagnostic approaches, but were diagnosed with lung cancer 231–1,954 days later (Supplementary Table 3). These individuals had median GEMINI scores of 0.78, significantly higher than those of individuals without cancer (Wilcoxon rank sum test, *P* = 0.0005) (Fig. 4b). Of these seven individuals, six had a score above the threshold at an 80% specificity, with the time to lung cancer diagnosis ranging from 231 to 1,868 days, suggesting that abnormalities in cfDNA mutational profiles could be detected years before standard diagnoses. Of these patients, five were ultimately diagnosed with non-small cell lung cancer (NSCLC) (two patients had stage I disease, one patient had stage III disease and stage information was unavailable for the other two patients), one patient was diagnosed with SCLC (stage unavailable) and the other patient for whom we do not have stage or histology information died within a few months of their diagnosis. The patient who was not detected by GEMINI had the longest time from blood draw to diagnosis (1,954 days). Interestingly, at the time of the initial blood draw, cancer was not suspected for four of these patients based on CT imaging and no biopsy was performed. For the remaining three patients, there was suspicion of cancer based on CT imaging and the patients underwent biopsy; however, their pathology report indicated a benign lung nodule, highlighting the limitations of current diagnostic approaches.

### Combining GEMINI with DELFI improves lung cancer detection

**Extended Data Fig. 8:**
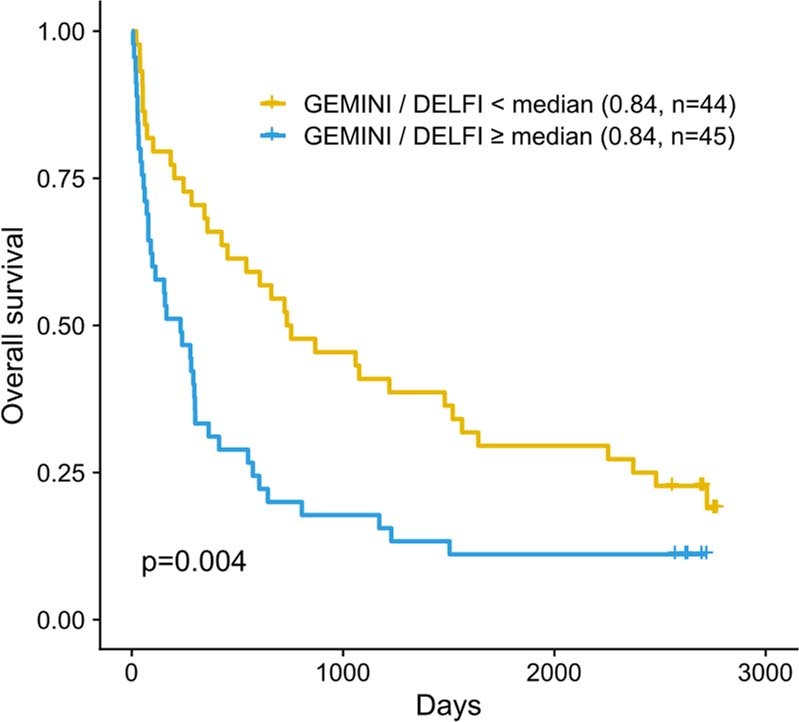
GEMINI / DELFI score and clinical outcome in lung cancer patients. Patients with lung cancer in the high-risk LUCAS cohort (n = 89) were stratified in two groups based on the median GEMINI / DELFI score among lung cancer patients of 0.84. Patients with a GEMINI / DELFI score ≥0.84 (yellow) had a significantly worse overall survival compared to patients with a GEMINI / DELFI score < 0.84 (blue) (p = 0.004, Log-rank test).

We examined whether GEMINI could be combined with DELFI, which uses cfDNA fragmentation features to improve detection of early stage lung cancer. Although GEMINI and DELFI scores were positively correlated (Spearman’s rho, 0.50; *P* < 0.0001), several samples that were missed by either approach in isolation were detected using a combined cross-validated GEMINI–DELFI approach (Methods), reducing false negatives by 56% at 80% specificity (Supplementary Fig. 13). The combined approach had higher overall performance, with an AUC of 0.93 (95% CI, 0.89–0.97) (*P* < 0.05 compared to GEMINI or DELFI alone) (Fig. 4d). For stage I patients (*n* = 13), DELFI or GEMINI alone achieved AUCs of 0.73 (95% CI, 0.59–0.88) and 0.80 (95% CI, 0.67–0.93), respectively, and an AUC of 0.87 (95% CI, 0.76–0.98) when combined (*P* < 0.05 compared to DELFI alone) (Fig. 4f). The combined approach provided an overall sensitivity of 91% at a specificity of 80% (GEMINI–DELFI score > 0.38) (Table 1). In principle, an initial blood-based test could increase adherence to lung cancer screening and reduce the number of unnecessary follow-up diagnostic approaches to identify individuals with cancer^15,18^. A positive blood test would subsequently be followed by standard LDCT imaging, thereby reducing harm from a false-positive blood test given that, currently, all individuals at high risk for lung cancer are recommended to receive LDCT^3^. When considering this approach as a pre-screen to LDCT, the sensitivity of the combined approaches would be >90% at a specificity of 85% (Table 1). Importantly, individuals with lower GEMINI–DELFI scores had better prognoses than individuals with higher scores (log-rank test, *P* = 0.004) (Extended Data Fig. 8), reducing the concern of false negatives with this approach, as individuals with lower scores would have a better prognosis and tumors could be detected in subsequent screens.

**Table 1:** Sensitivity of GEMINI and DELFI followed by LDCT for lung cancer detection

	GEMINI	GEMINI, DELFI	GEMINI, DELFI, LDCT
Overall (*n* = 89)	76%	91%	93%
Overall blended (*n* = 89)	69%	88%	91%
Stage			
Stage I (*n* = 13)	62%	85%	88%
Stage II (*n* = 7)	86%	86%	95%
Stage III (*n* = 30)	73%	90%	95%
Stage IV (*n* = 39)	82%	95%	93%
Histology			
Adenocarcinoma (*n* = 45)	73%	89%	93%
Squamous (*n* = 24)	71%	88%	91%
Small cell (*n* = 10)	100%	100%	95%

Sensitivities were determined at specificities of 80% for GEMINI or GEMINI and DELFI, or at a combined specificity of 85% for GEMINI, DELFI and LDCT with a GEMINI–DELFI pre-screen specificity of 62%. DELFI had an overall sensitivity of 80% at 80% specificity. Based on the high-risk subset of patients analyzed from the LUCAS cohort^18^, LDCT in this setting had a specificity of 66% and an assumed sensitivity of 95%. Overall blended sensitivity reflects the sensitivity expected in a screening population weighted by the proportion of lung cancers detected in the National Lung Screening Trial population at each stage^5^.

### Validation of GEMINI models

To validate the cross-validated GEMINI and combined cross-validated GEMINI–DELFI models, we evaluated an additional cohort of individuals from lung cancer screening programs (*n* = 57; Supplementary Table 6). This cohort included asymptomatic high-risk individuals with predominantly early stage cancers (stage I, 32; stage II, 4; stage III, 3; stage IV, 2; and unknown, 1) as well as individuals without cancer (*n* = 15). Of 42 individuals with lung cancer, 21 (50%) were diagnosed with stage IA disease, similar to the proportion detected by LDCT in the National Lung Screening Trial^5^. We isolated cfDNA from the plasma of these individuals and performed WGS with coverage and feature metrics similar to the LUCAS cohort (Supplementary Fig. 14). We analyzed these samples using the fixed GEMINI and fragmentation machine-learning models from the LUCAS cohort analyses. Consistent with our initial studies, GEMINI scores were higher in high-risk individuals (aged 50–80 years with a smoking history) with cancer compared to those without cancer (Wilcoxon rank sum test, *P* = 0.001) (Fig. 4c). Across the validation and LUCAS cohorts, GEMINI scores of patients with later stage lung cancer (stages III and IV, median GEMINI score of 0.74) were significantly higher than those with early stage cancer (stages I and II, median GEMINI score of 0.64) (Wilcoxon rank sum test, *P* = 0.03). The GEMINI score threshold corresponding to 80% specificity from the LUCAS cohort analyses resulted in a specificity of 86% (95% CI, 57–98%) in the validation cohort. The performance of GEMINI for detecting stage I disease in this cohort was high, with an overall AUC of 0.81 (95% CI, 0.67–0.94) and 0.86 (95% CI, 0.74–0.97) when combined with DELFI (Fig. 4f). Overall, these analyses suggest that genome-wide mutational profiling is generalizable for detection of early stage lung cancer in high-risk populations.

### GEMINI scores associate with smoking history

**Extended Data Fig. 9:**
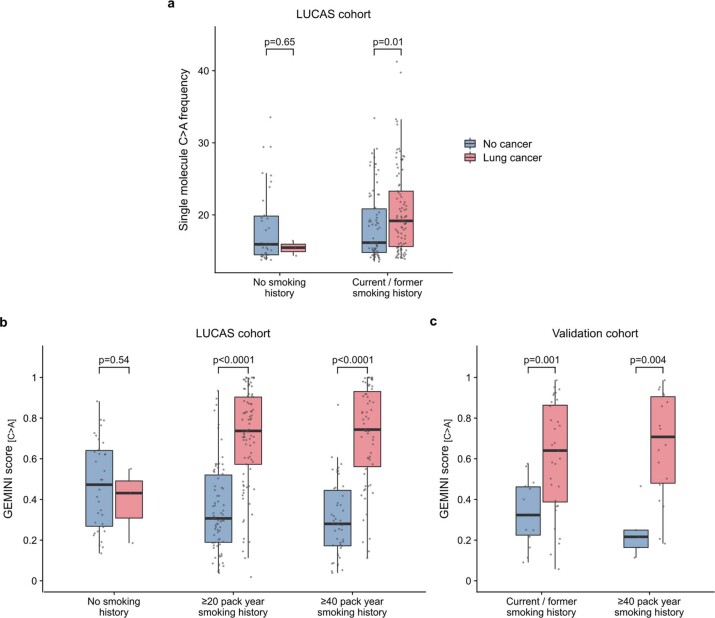
GEMINI scores and smoking exposure in lung cancer patients. **a**, Single molecule C > A frequencies were similar in never smokers with lung cancer (n = 3) or without lung cancer (n = 34) in the LUCAS cohort. In current or former smokers in the high-risk group, with a ≥20 pack year smoking history and age 50–80, the single molecule C > A frequencies were slightly higher in individuals with lung cancer (n = 89) compared to individuals without lung cancer (n = 74). **b**, GEMINI scores were similar in never smokers with lung cancer (n = 3) or without lung cancer (n = 34). In the high-risk group, GEMINI scores were higher in individuals with lung cancer (n = 89) compared to those without lung cancer (n = 74). Similarly, for individuals with a ≥40 pack year smoking history and age 50–80, the GEMINI scores were higher in those with lung cancer (n = 63) compared to those without lung cancer (n = 46). **c**, GEMINI scores were higher in individuals with lung cancer in the validation cohort in current/former smokers age 50–80 with (n = 32) and without lung cancer (n = 14) and in the subset with a ≥40 pack year smoking history with (n = 18) and without lung cancer (n = 5). P-values in **a-c** were obtained from two-sided Wilcoxon rank sum tests. All boxplots represent the interquartile range with whiskers drawn to the highest value within the upper and lower fences (upper fence = 0.75 quantile + 1.5 × interquartile range; lower fence = 0.25 quantile – 1.5 × interquartile range). The solid middle line in the boxplot corresponds to the median value.

As somatic mutations in lung cancer in smokers are related to smoking, we reasoned that there would be a relationship between GEMINI scores and smoking history. Although overall cfDNA C>A mutation frequencies were similar among non-smokers with and without lung cancer (Wilcoxon rank sum test, *P* = 0.65), smokers with lung cancer had higher overall mutation frequencies than smokers without cancer (Wilcoxon rank sum test, *P* = 0.01) and dramatically higher GEMINI scores (Wilcoxon rank sum test, *P* < 0.0001) (Extended Data Fig. 9a,b). The GEMINI score was positively associated with years of smoking among patients with cancer (Spearman’s rho, 0.24; *P* = 0.01). Interestingly, in individuals without cancer, the GEMINI score was negatively correlated with smoking exposure (Spearman’s rho, −0.25; *P* = 0.002), potentially reflecting smoking-related DNA damage in non-cancer tissues^37^ that may contribute to alterations of cfDNA. Analyses of patients in the LUCAS and validation cohorts suggested that GEMINI may have higher performance in detecting individuals with greater smoking history (Fig. 4e,f and Extended Data Fig. 9b,c), including an increase in performance in the LUCAS cohort to an AUC of 0.90, and to an AUC of 0.95 with the combined GEMINI–DELFI approach (DeLong’s test, *P* < 0.05 compared to DELFI alone, which had an AUC of 0.88). A positive GEMINI test at a specificity of 80% was associated with a 13.5-fold increase in the odds of cancer among ≥20 pack-year smokers (95% CI for odds ratio, 6.7–30.7; *P* < 0.0001), and with a 20.1-fold increase among ≥40 pack-year smokers (95% CI for odds ratio, 7.7–54.6; *P* < 0.0001). These observations suggest that smoking exposure results in sequence alterations in both ctDNA and non-tumor cfDNA, affecting distinct genomic regions that may facilitate cancer detection using GEMINI.

### GEMINI can distinguish between histological subtypes of lung cancer

**Fig. 5:**
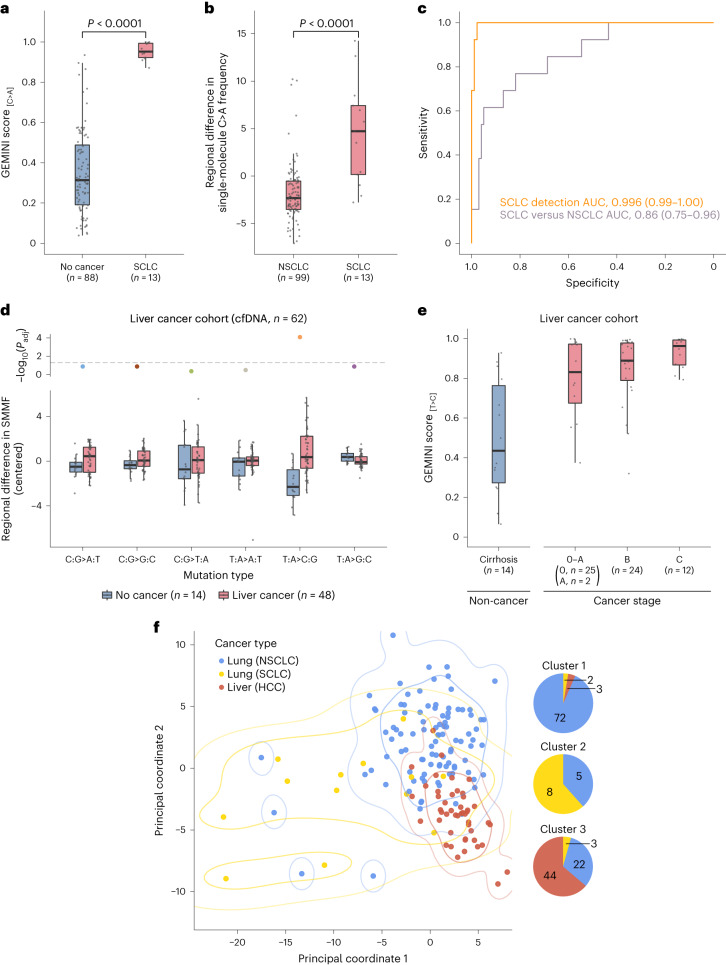
GEMINI approach for non-invasive detection across multiple cancer types. **a**, GEMINI scores in patients with SCLC and high‐risk individuals without cancer in the LUCAS and validation cohorts show high performance for detecting cancer (two-sided Wilcoxon rank sum test, *P* < 0.0001). **b**, Regional differences in single-molecule C>A frequency in the LUCAS and validation cohorts demonstrate that GEMINI can be used to identify the bins that are most altered between SCLC and NSCLC (two-sided Wilcoxon rank sum test, *P* < 0.0001). **c**, ROC curves for the detection of SCLC (*n* = 13) compared to non‐cancer controls (*n* = 88) (orange) as well as for distinguishing SCLC (*n* = 13) from NSCLC (*n* = 99) (purple) in the combined LUCAS and validation cohorts. **d**, Cross-validated regional differences in SMMFs in cfDNA in the liver cancer cohort, median-centered within each mutation type, show a high level of T>C mutations in patients with HCC. Adjusted *P* values (*P*_adj_) were generated using the two-sided Wilcoxon rank sum test and were corrected for multiple comparisons using the Benjamini–Hochberg method. The horizontal dashed line indicates a *P* value of 0.05. **e**, GEMINI scores in the liver cancer cohort with the number of individuals indicated at each stage demonstrate high sensitivity for detection of liver cancer across all stages. **f**, Principal coordinate analysis of the Euclidean distance matrix reflecting cross-validated pairwise differences in regional mutation frequencies between NSCLC, SCLC and HCC. The first two principal coordinates are shown with contours indicating kernel density estimations for 0.7 and 0.95 probability for each cancer type. The composition of cancer types in clusters derived from *K*-means clustering with *k* = 3 is indicated to the right. All boxplots represent the interquartile range, with whiskers drawn to the highest value within the upper and lower fences (upper fence, 0.75 quantile + 1.5× interquartile range; lower fence, 0.25 quantile – 1.5× interquartile range). The solid middle line in the boxplot corresponds to the median value.

Given the important differences between biological features and clinical management of SCLC and NSCLC, we examined whether genome-wide mutational profiles could be used to detect SCLC and to non-invasively distinguish this cancer from NSCLC. GEMINI scores were extremely high in patients with SCLC (*n* = 13) compared to those in individuals without cancer (*n* = 88) (Wilcoxon rank sum test, *P* < 0.0001) (Fig. 5a and Supplementary Tables 3 and 6) and could distinguish among these with an AUC of >0.99 (95% CI, 0.99–1.00) (Fig. 5c). We used GEMINI to assess regional mutation differences in cfDNA of patients with SCLC compared to those with NSCLC (*n* = 99) and found that mutation frequencies obtained in this way were higher in SCLC (Wilcoxon rank sum test, *P* < 0.0001) (Fig. 5b and Supplementary Table 7) and could be used distinguish this cancer type from NSCLC (AUC, 0.86; 95% CI, 0.75–0.96) (Fig. 5c). These findings suggest that genome-wide mutation profiles may provide a non-invasive approach for detecting SCLC and distinguishing lung cancers with different histological subtypes.

### Detection of other cancer types with GEMINI

To explore the generalizability of GEMINI to detect other cancers, we applied the method to a prospective cohort of individuals with or without liver cancer (*n* = 62; Supplementary Table 8). Cross-validated regional differences in mutation frequencies identified a significant difference in genome-wide T>C mutation profiles (Fig. 5d) in individuals with liver cancer. The derived GEMINI scores were higher in individuals with liver cancer across all stages (0–A, B and C) compared to those with cirrhosis (*P* < 0.01 for each comparison) (Fig. 5e). Similar to analyses of patients with lung cancer, GEMINI scores from patients with liver cancer were generally related to ctDNA levels, increasing with tumor fraction estimates^35^ (Wilcoxon rank sum test, *P* = 0.008) (Supplementary Fig. 12b).

As cfDNA mutation profiles appeared cancer type-specific, we reasoned that GEMINI could distinguish among different cancer types. We compared cfDNA mutation profiles between NSCLC, SCLC and hepatocellular carcinoma (HCC) (*n* = 159) and found that they largely clustered into three groups, with each cancer type comprising the majority of observations in a cluster (Fig. 5f and Methods). Exclusion of the most common tumor-specific alterations (Fig. 3a,b and Extended Data Fig. 1) prevented accurate grouping by cancer type (Supplementary Fig. 15). Overall, these analyses suggest that mutation profiles may be useful for non-invasive determination of cancer origin.

### GEMINI scores reflect ctDNA burden during therapy

**Extended Data Fig. 10:**
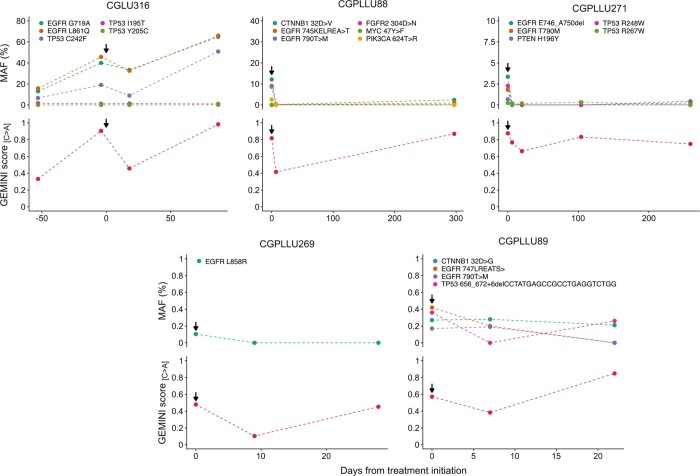
GEMINI scores and MAF levels during therapy. Individuals with a smoking history as well as availability of targeted deep sequencing^11^ and low coverage WGS data^13^ were analyzed before and during treatment with tyrosine kinase inhibitors (arrows indicate initiation of treatment). GEMINI scores were associated with the median mutant allele fraction (MAF) of detectable mutations at each timepoint with values of zero used in CGPLLU269 samples where no mutations were detected (Spearman’s correlation coefficient = 0.53, p = 0.02, two-sided). The range of median MAFs for all GEMINI positive patients was 0.17% to 50.91% at 80% specificity.

To explore whether GEMINI could be used to monitor patients during therapy, we assessed serial blood samples from patients with lung cancer who were undergoing treatment with EGFR or ERBB2 inhibitors with mutant allele fractions (MAFs) as low as 0.1% (Supplementary Table 5). Using the fixed lung cancer model, we found that after the initiation of therapy, GEMINI scores decreased in all patients, consistent with an initial response to therapy, and that over time, GEMINI scores increased, consistent with the known progression of these individuals (Extended Data Fig. 10). GEMINI scores were positively correlated with MAFs from targeted sequencing of these patients (Spearman’s correlation coefficient, 0.53; *P* = 0.02), indicating that GEMINI has a high sensitivity to low MAF levels and reflects ctDNA burden during therapy.

We used MAF values from these samples to gain insight into the limit of detection (LOD) of GEMINI. GEMINI-positive samples had median MAFs ≥ 0.17%, and in silico dilutions of these samples as well as PCAWG tumors at known concentrations with healthy cfDNA resulted in a high sensitivity at low tumor fractions previously observed for early stage lung cancers^8,10,15,38,39^. These analyses suggest a LOD of ~0.1% using low-coverage WGS and potentially lower LODs at deeper sequencing levels (for example, 8×) (Supplementary Fig. 16, Supplementary Note and Supplementary Table 9).

## Discussion

Here, we show that cancer can be detected non-invasively through single-molecule mutation profiles obtained from low-coverage WGS of cfDNA. Tumor type-specific mutational landscapes were detectable in plasma from patients with cancer and appear to be related to replication timing and other chromatin features in which repair of DNA damage may be impaired^40^. The method described here does not require deep sequencing of matched blood cells to filter hematopoietic alterations^16^ or tumor sequencing to identify tumor-specific mutations to evaluate in the plasma^22^, and therefore the approach is amenable for de novo detection and characterization of cancer. GEMINI involves the construction of a single genomic library from cfDNA followed by light WGS, which may provide more information and have practical advantages compared to approaches based on more complex methods that target a small subset of the genome^8,10,15^, measure different analytes^10^ or involve extensive sample processing such as bisulfite conversion or immunoprecipitation^14,38,41^. The combination of genome-wide GEMINI mutational and DELFI fragmentation analyses of cfDNA may provide an opportunity for the cost-efficient and scalable detection of cancer.

Although many patients in this study were at risk for developing cancer, our validation cohorts were small. Large-scale analytical and clinical validation of performance, including more precise determination of sensitivity, specificity and detection limits in asymptomatic screening populations for lung, liver or other cancers are needed before clinical use. It will be important to also consider the risks and benefits associated with a blood-based pre-screen prior to and in conjunction with other screening approaches, such as LDCT, in large-scale prospective studies. Sequencing the genome at higher coverage using new sequencing approaches^42^, as well as advances in reducing errors during library preparation and next-generation sequencing would be expected to further lower the LOD of GEMINI, which may be necessary to detect tumors that shed very low amounts of cfDNA^15,43^. As mutation rates vary substantially across cancer genomes^31^, detection of altered regional mutational frequencies in cfDNA provides a generalizable approach for cancer detection and monitoring.

## Methods

### Study populations

The collection of patient samples for this study conformed to all relevant ethical regulations. Collection protocols were approved by the Danish Regional Ethics Committee and the Danish Data Protection Agency (LUCAS cohort), the Human Research Protection Office for the Department of Defense (Detection of Early Lung Cancer Among Military Personnel (DECAMP) samples), the Allegheny Health Network (AHN) Institutional Review Board (AHN samples) and the Johns Hopkins Institutional Review Board (liver cancer cohort). All patients provided written informed consent and the studies were performed according to the Declaration of Helsinki.

Tissue samples from the PCAWG Consortium consisted of 2,778 tumors with somatic mutation calls^25^. Hypermutated tumors, including those with putative polymerase epsilon or mismatch-repair defects, as well as one tumor with temozolomide treatment, were excluded from analysis (*n* = 49), as well as cancer types with less than 20 samples (*n* = 129 samples) and cancer types with an average of <250 mutations per sample (pilocytic astrocytoma, *n* = 89 samples) resulting in 2,511 tumors across 25 common cancer types. Single-molecule mutation analyses were performed on lung cancer and matched solid tissue or blood cells from 86 donors who passed quality-control metrics, 65 of whom had mutations attributed to smoking-related signature 4 (ref. ^25^). Of these 65 patients, 31 had both tumor tissue and blood-derived normal sequencing data available. Additional information regarding these samples is available in Supplementary Table 1 and at https://dcc.icgc.org/releases/PCAWG.

The LUCAS cohort^18^ was a prospectively collected group of 365 patients that presented in the Department of Respiratory Medicine, Infiltrate Unite, Bispebjerg Hospital, Copenhagen, Denmark, with a positive imaging finding on a chest X-ray or a chest CT (Supplementary Table 3). The high-risk LUCAS cohort was defined as individuals at high risk for lung cancer (aged 50–80 years with a ≥20 pack-year smoking history) and included individuals with primary lung cancer at baseline (*n* = 89) as well as individuals without prior, baseline or future cancer (*n* = 74).

The validation cohort comprised individuals from lung cancer screening programs (*n* = 57) (Supplementary Table 6), including asymptomatic high-risk individuals with predominately early stage cancers or nodules determined to be benign that had a liquid biopsy collected before a possible diagnosis of lung cancer. Individuals were enrolled through either the DECAMP-1 protocol^44^ or through screening efforts at the AHN. The DECAMP-1 protocol included current or former cigarette smokers with ≥20 pack-year exposure and radiological findings indicating an indeterminate pulmonary nodule of 0.7 to 3.0 cm in size identified within 12 months prior to enrollment with an additional CT scan within 3 months prior to enrollment. Individuals enrolled at the AHN were identified based on eligibility for high-risk screening for lung cancer using low-dose helical CT scanning or an indication for lung cancer screening based on other high-risk characteristics, such as a family history of lung cancer.

The lung cancer monitoring cohort consisted of serial blood draws from a cohort of patients with lung cancer that were undergoing treatment with EGFR or ERBB2 inhibitors^11^. The study population included samples from serial blood draws (*n* = 18) from patients with a smoking history (*n* = 5) with both targeted sequencing and WGS available^13^. Additional information regarding these samples is available in Supplementary Table 5.

The liver cancer cohort consisted of 62 patients with either liver cancer (*n* = 48) or cirrhosis (*n* = 14). Samples were collected prospectively as part of the HCC Biomarker Registry at the Johns Hopkins University School of Medicine, Baltimore, Maryland, USA. Liver cancer was confirmed by appropriate imaging characteristics as defined by accepted guidelines. Tumor staging was determined by the Barcelona Clinic Liver Cancer staging system. Detailed clinical data were extracted from electronic medical records. Additional information regarding these samples is available in Supplementary Table 8.

A previously published lung cancer cohort^18^ was not used in this study as it included samples from sources that did not collect information related to smoking exposure.

### Blood sample collection and preservation

The sample collection for the LUCAS cohort was performed at the time of the screening visit, and venous peripheral blood was collected in one K2-EDTA tube. Within 2 h of blood collection, tubes were centrifuged at 2,330×*g* at 4 °C for 10 min.

For the validation cohort, venous peripheral blood from each individual was collected in one K2-EDTA tube (AHN) or one Streck tube (DECAMP). Tubes from the AHN and the DECAMP collections were centrifuged at low speed (800–1,600×*g*) for 10 min; the plasma portion was spun a second time for 10 min.

For the lung cancer monitoring cohort, whole blood was collected in EDTA tubes and processed immediately or within 1 day after storage at 4 °C, or was collected in Streck tubes and processed within 2 days of collection as previously described^13^. Plasma and cellular components were separated by centrifugation at 800×*g* for 10 min at 4 °C. Plasma was centrifuged a second time at 18,000×*g* at room temperature (18–24 °C) to remove any remaining cellular debris.

For the liver cancer cohort, venous peripheral blood was collected in one K2-EDTA tube. Within 2 h of blood collection, tubes were centrifuged at 2,330×*g* at 4 °C for 10 min, plasma was transferred to new tubes and the samples were spun at 18,000×*g* for 10 min at room temperature to pellet any remaining cellular debris. In all cases, after centrifugation, plasma samples were aliquoted and stored at −80 °C.

### Plasma sequencing library preparation

The cfDNA was isolated from 2–4 ml of plasma using the Qiagen QIAamp Circulating Nucleic Acids Kit, eluted in 52 μl of RNase-free water containing 0.04% sodium azide (Qiagen) and stored in LoBind tubes (Eppendorf) at −20 °C. The concentration and quality of cfDNA were assessed using the Bioanalyzer 2100 (Agilent Technologies).

Next-generation sequencing cfDNA libraries were prepared for WGS using 15 ng of cfDNA when available, or the entire purified amount when less than 15 ng was available. In brief, genomic libraries were prepared using the NEBNext DNA Library Prep Kit for Illumina (New England Biolabs) with four main modifications to the manufacturer’s guidelines: (1) the library purification steps use the on-bead AMPure XP (Beckman Coulter) approach to minimize sample loss during elution and tube transfer steps; (2) NEBNext End Repair, A-tailing and adaptor ligation enzyme and buffer volumes were adjusted as appropriate to accommodate on-bead AMPure XP purification; (3) Illumina dual index adaptors were used in the ligation reaction; and (4) cfDNA libraries were amplified with Phusion Hot Start Polymerase. All samples underwent a four-cycle PCR amplification after the DNA ligation step.

### WGS and preprocessing of sequencing data

Tissue sequencing data from PCAWG samples were obtained as Binary Alignment Map files that were indexed using SAMtools (v.1.9)^45^. Libraries prepared from cfDNA from patients with cancer and from cancer-free individuals were sequenced at ~2× coverage per sample using 100 bp paired-end runs (200 cycles) on the Illumina HiSeq 2000/2500 (LUCAS^18^, validation and lung cancer monitoring cohorts^13^) and the NovaSeq 6000 (liver cancer cohort). To assess concordance between tissue and cfDNA mutation profiles in cancer types with few available samples, we re-sequenced LUCAS samples from patients with melanoma (*n* = 2) and lymphoma (*n* = 1) as well as 40 control individuals without cancer and 15 individuals with largely advanced lung cancers to a median of 10× coverage on the Illumina NovaSeq 6000 (Supplementary Table 4). Before alignment, adaptor sequences were filtered from reads using fastp (≥0.20.0)^46^. Sequence reads were aligned against the hg19 human reference genome using Bowtie2 (v.2.3.5.1)^47^, and duplicate reads were removed using Sambamba (≥0.7.1)^48^. Sequencing data metrics are reported in Supplementary Tables 3–6 and 8.

### Downsampling and dilution of somatic mutations from PCAWG lung cancer samples

The downsampling and dilution experimental methodology is shown in Supplementary Fig. 2. Specifically, somatic mutation calls (*n* = 3,393,564 mutations) were obtained for individuals in PCAWG with lung cancer with the presence of signature 4 (*n* = 65)^26^. We excluded mutations with a missing value for either the number of reference or mutant alleles observed (*n* = 5,857), resulting in 3,387,707 somatic mutations across 65 individuals. For a given individual, we considered each observation of the reference or mutant allele separately. We first computed the number of sequenced observations that were tumor-derived as the total number of observations multiplied by the tumor purity of the sample. We then spiked in observations with the reference allele until 10^−1^, 10^−2^, 10^−3^ or 10^−4^ of the observations were of tumor origin. We next computed the average coverage of mutated positions following dilution and randomly sampled the observations to achieve a desired coverage of 8×, 4×, 2×, 1× and 0.5×. For each known somatic mutation in an individual’s cancer genome, we tallied the number of times that we observed the mutation for each combination of dilution amount and genome coverage, and used this information to compute the percent of mutations observed in single DNA molecules.

### Identification of single and doublet base changes in single molecules

We scanned the primary alignment of properly paired read pairs that mapped to autosomes in non-overlapping 100 kb bins and obtained the base call, Phred score and mapping quality of each sequenced base using pysam (v.0.16.0.1). We considered only read pairs with a MAPQ of ≥40 and only positions within each read with a Phred score of ≥30. To avoid counting larger sequence changes, we retained alterations where the adjacent bases were identical to the reference allele and had Phred scores of ≥30. In addition, we removed positions that overlapped the Duke Excluded Regions track (http://hgdownload.cse.ucsc.edu/goldenpath/hg19/encodeDCC/wgEncodeMapability). In each 100 kb bin, we counted the number of sequenced bases that were C:G or A:T in the reference genome. We also counted the number of times that we observed each type of single-base change (C:G>A:T, C:G>G:C, C:G>T:A, T:A>A:T, T:A>C:G and T:A>G:C) and CC:GG>AA:TT doublet base changes in 100 kb bins. We counted observations separately based on whether the purine or the pyrimidine of each base pair was in read 1 or read 2 of the paired-end sequencing data. To exclude potential germline variants, we used the gnomAD database (v.3.0), which contains genetic variants from >70,000 whole genomes^49^. We removed any candidate variants if the variant was present in gnomAD but the variant did not pass gnomAD quality filters, or if the variant was present in gnomAD with an allele frequency of >1 in 100,000. For PCAWG samples, we annotated the remaining variants in each sample, indicating whether they were called as a somatic or germline variant by the PCAWG consortium. For analyses of tissue samples, we considered variants observed at any position in a fragment. For plasma samples, we analyzed positions in fragments that were sequenced by both read 1 and read 2 of the read pair with the same base call. To account for potential differences in sequencing depth between samples, single-molecule mutation frequencies were always computed as the number of each sequence change divided by the number of evaluable bases, defined as the number of positions in fragments in which each sequence change could be detected after quality and germline filtering.

### Generation of regional differences in single-molecule mutation frequencies

The approach to compute the regional difference in single-molecule mutation frequency for a given mutation type is shown in Extended Data Fig. 5. Specifically, we first aggregated the 100 kb bins to 1,144 non-overlapping 2.5 Mb bins. Let ${y}_{i}^{0}$ and ${y}_{i}^{1}$ denote the number of sequence changes (for example, C>A) at bin *i* for a participant without and a participant with cancer, respectively. We denote the corresponding number of evaluable positions (for example, number of C:G bases that pass quality filters) by ${x}_{i}^{0}$ and ${x}_{i}^{1}$. The difference in the number of sequence changes at bin *i* relative to the number of evaluable bases comparing participants with and without cancer for a training set comprising *n* − 1 samples with *J* participants with cancer and *K* participants without cancer (*J* + *K* = *n* − 1) is given by:

$${\delta }_{i}=\frac{{\sum }_{j}{y}_{{ij}}^{1}}{{\sum }_{j}{x}_{{ij}}^{1}}-\frac{{\sum }_{k}{y}_{{ik}}^{0}}{{\sum }_{k}{x}_{{ik}}^{0}}{\rm{for}}\,i=1,\ldots ,1,144.$$

Let δ_(s)_ denote the *s*^th^ order statistic such that δ_(1)_ is the bin most depleted for sequence changes in cancers relative to non-cancers and δ_(1,144)_ is the bin most enriched for sequence changes in cancers relative to non-cancers. Feature selection in the training set proceeds by identifying the bins at the bottom decile of δ (bins with values ${\delta }_{(1)},\ldots ,{\delta }_{\left(114\right)}$) and the bins at the top decile (bins with values ${\delta }_{(1,144)},\ldots ,{\delta }_{\left(1,030\right)}$). Denoting the bin sets for the bottom and top deciles by {*A*_−*h*_} and {*B*_−*h*_}, respectively, for a training set that excludes the *h*^th^ sample, the regional difference in single-molecule mutation frequency for the test sample is given by:

$${{\rm{regional}}\; {\rm{difference}}}_{h}=\frac{{\sum }_{b\in \left\{{B}_{-h}\right\}}{y}_{{bh}}}{{\sum }_{b\in \left\{{B}_{-h}\right\}}{x}_{{bh}}}-\frac{{\sum }_{a\in \left\{{A}_{-h}\right\}}{y}_{{ah}}}{{\sum }_{a\in \left\{{A}_{-h}\right\}}{x}_{{ah}}}$$

Using leave-one-out cross validation, we repeated this procedure such that every participant appeared in the test set once and the regional differences in single-molecule mutation frequency was obtained for all *n* participants.

### Downsampling the regional difference in single-molecule C>A frequency to 1× coverage in PCAWG

For brevity, we use the alternative notation for the regional difference $\frac{{y}_{{Bh}}}{{x}_{{Bh}}}-\frac{{y}_{{Ah}}}{{x}_{{Ah}}}$, where ${y}_{{Bh}}=\sum _{b\in \left\{{B}_{-h}\right\}}{y}_{{bh}}$. Denoting the down-sampled (*) regional difference by ${{\rm{regional\; difference}}}_{h}^{* }=\frac{{y}_{{Bh}}^{* }}{{x}_{{Bh}}^{* }}-\frac{{y}_{{Ah}}^{* }}{{x}_{{Ah}}^{* }}$, we derived these quantities first by determining the number of evaluable C:G positions in the hg19 reference genome, *r*_*A*_ and *r*_*B*_. Next, we randomly sampled (without replacement) *r*_*A*_ indices from the set $\left\{1,\ldots {,x}_{{Ah}}\right\}$ and *r*_*B*_ indices from the set $\left\{1,\ldots {,x}_{{Bh}}\right\}$ to represent indices of evaluable positions in these bin sets. The number of indices in the two random samples that were less than or equal to *y*_*Ah*_ and *y*_*Bh*_ were used for ${y}_{{Ah}}^{* }$ and ${y}_{{Bh}}^{* }$, respectively. The above procedure was repeated until all participants in PCAWG had a down-sampled regional difference in the single-molecule C>A frequency.

### Generation of GEMINI scores

To provide a calibrated score that captures the relationship between the regional difference in single-molecule C>A frequency and the probability that an individual has lung cancer in the high-risk LUCAS cohort, we fit a logistic regression model for cancer status (lung GEMINI model) using the regional difference in single-molecule C>A frequency as a covariate and extracted the fitted probability of cancer for each individual (lung GEMINI score). In addition, we generated lung GEMINI scores for the validation cohort, the cohort of patients with a baseline negative test that later developed lung cancer, the cohort of patients with lung cancer that were monitored during therapy as well as the remaining samples in the LUCAS cohort using the fixed bin sets and lung GEMINI model. For the liver cancer cohort, GEMINI scores were generated by fitting a logistic regression model for cancer status (liver GEMINI model) using the regional difference in single-molecule T>C frequency as the covariate and extracting the fitted probability of cancer for each individual (liver GEMINI score).

### Generation of DELFI and combined GEMINI–DELFI scores

To evaluate whether fragmentation features could further improve the prediction of cancer status by GEMINI, we used the approach previously described^18^ on the same training sets used to generate cross-validated GEMINI scores. In brief, we tiled the hg19 reference genome into non-overlapping 5 Mb bins. Bins with an average GC content <0.3 and an average mappability <0.9 were excluded, leaving 473 bins spanning approximately 2.4 Gb of the genome. Fragment size analyses were conducted on fragments with a mapping quality of at least 30. Ratios of the number of short (100–150 bp) to long (151–220 bp) fragments across the 473 bins were normalized for GC content and library size as previously described^18^. For each training set, we performed a principal component analysis on the fragmentation profiles and retained the minimum number of principal components needed to explain 90% of the variance between participants. Chromosomal arm copy number was summarized by computing a *z*-score for each arm using an expected coverage and standard deviation computed from an external reference set of 54 non-cancer controls (https://github.com/cancer-genomics/PlasmaToolsHiseq.hg19). The 39 *z*-scores and principal components were integrated as covariates in a logistic regression model with a LASSO penalty. To generate DELFI scores in the validation cohort, we used the model that was trained on a larger set of 158 non-cancers and 129 cancers^18^. The combined GEMINI–DELFI score was computed by averaging the individual GEMINI and DELFI scores for each patient.

### Generation of regional differences in C>A frequencies between SCLC and NSCLC

The regional differences in single-molecule C>A frequencies were computed as described above, in which individuals with SCLC were compared with those with NSCLC. To maximize the number of samples used for identifying bin sets A and B, we combined samples from the high-risk LUCAS cohort (*n* = 10 SCLC, *n* = 75 NSCLC) with individuals who were smokers and aged 50–80 years from the validation cohort (*n* = 3 SCLC, *n* = 24 NSCLC).

### Analysis of different tumor types

We computed the regional difference in single-molecule mutation frequency as described above by iteratively holding out each individual with either NSCLC, SCLC or HCC (*n* = 159) and identifying bin sets A and B using all other individuals. For each mutation type (C>A, C>G, C>T, T>A, T>C and T>G), individuals with NSCLC were compared to those with SCLC, individuals with NSCLC were compared to those with HCC and individuals with SCLC were compared to those with HCC, yielding 18 regional differences in mutation frequencies per individual. Principal coordinate analysis was performed on the similarity matrix generated from the Euclidean distance between pairwise samples using these 18 regional differences in mutation frequencies. *K*-means clustering was performed on the matrix of 18 regional differences in mutation frequencies with the number of clusters (*k*) set to three. As a negative control, principal coordinate analysis was also performed on a similarity matrix generated from the Euclidean distance between pairwise samples after excluding C>A and T>C mutations that were most frequently observed in lung and liver cancers.

### Association of mutation frequencies with genomic features

Replication timing tracks, computed by averaging the wavelet-smoothed transform of the six fraction profile, representing different time points during replication in 1 kb bins were downloaded from the University of California, Santa Cruz Genome Browser from IMR90, NHEK and GM12878 cell lines^50,51^. We computed the weighted average in each 2.5 Mb bin, with higher values indicating earlier replication timing. Gene expression values were obtained as raw counts using recount3 (v.1.0.2)^52^ and converted to transcripts per million from lung adenocarcinoma (*n* = 542), lung squamous cell carcinoma (*n* = 504), melanoma (*n* = 472) and B cell non-Hodgkin lymphoma (*n* = 48) generated by The Cancer Genome Atlas. For each cancer type, we first averaged transcript per million values for each gene across samples. The gene expression in each 2.5 Mb bin in each cancer type was computed as the sum of the transcripts per million overlapping each bin weighted by the length of the transcript. These values were then averaged between lung adenocarcinoma and lung squamous cell carcinoma to obtain a single lung cancer gene expression estimate in each bin. A/B compartmentalization data generated at 100 kb resolution through eigenvector analysis of 450 K methylation array data was obtained for 12 cancer types and through eigenvector analysis of Hi-C data for GM12878 cells^33^. The weighted average of the eigenvectors in 100 kb bins was computed for each 2.5 Mb bin. The average of these values from lung adenocarcinoma and lung squamous cell carcinoma was used for lung cancer analyses, GM12878 was used for B cell non-Hodgkin lymphoma analyses and the average across all 12 cancer types was used for melanoma analyses in the absence of skin A/B compartmentalization data. ChIP–seq data for H3K9me3 of A549 cells (three pooled replicates), GM23248 cells and Karpas 422 cells (two pooled replicates) represented as the fold change of coverage in enriched samples with respect to control samples was downloaded from the ENCODE portal^53^ for analyses of NSCLC, melanoma and B cell non-Hodgkin lymphoma, respectively. The weighted average of the fold changes was computed in each 2.5 Mb bin for each cell type. GC content in each 2.5 Mb bin was obtained from the hg19 reference genome. Mappability, reflecting how uniquely 100-mer sequences align to a region of the genome, was downloaded (http://hgdownload.cse.ucsc.edu/goldenpath/hg19/encodeDCC/wgEncodeMapability/wgEncodeCrgMapabilityAlign100mer.bigWig) and aggregated into 2.5 Mb bins as the weighted average of mappability scores overlapping each bin. Genome-wide copy number was estimated for each sample using ichorCNA (v.0.3.2). Average copy number per genomic bin was computed as the weighted average of the copy number in segments overlapping each bin.

### Estimation of the fraction of tumor DNA in plasma

The percentage of tumor DNA in plasma was estimated for samples in the LUCAS and liver cancer cohorts using ichorCNA^35^.

### Estimation of 8-oxo-dG levels

The 8-oxo-dG level was estimated for each sample as the ratio of single-molecule C>A frequencies when guanine or G>T was on read 1 and cytosine or C>A was on read 2 to when cytosine or C>A was on read 1 and guanine or G>T was on read 2. These data are reported in Supplementary Tables 1, 3–6 and 8.

### Statistics

The Wilcoxon rank sum test was used to generate *P* values for two-group comparisons. Correlation analysis of continuous variables was performed using either Pearson’s product-moment correlation coefficient or Spearman’s rank correlation coefficient. All *P* values were based on two-sided hypothesis tests unless otherwise specified. Receiver operator characteristic curves were compared using DeLong’s test. All confidence intervals for area under the receiver operator characteristic curve indicate a confidence level of 95% and were based on DeLong’s method. CIs for coefficients in logistic regression models assume normality and were indicated at a 95% confidence level. CIs for specificity estimates were based on a binomial model and were indicated at a 95% confidence level. Analyses were performed with R (v.3.6.1 and later versions) and Python (v.3.8.2).

### Reporting summary

Further information on research design is available in the Nature Portfolio Reporting Summary linked to this article.

## Online content

Any methods, additional references, Nature Portfolio reporting summaries, source data, extended data, supplementary information, acknowledgements, peer review information; details of author contributions and competing interests; and statements of data and code availability are available at 10.1038/s41588-023-01446-3.

### Supplementary information

Supplementary Information Supplementary Note, Supplementary Figs.1–16.

Reporting Summary

Supplementary Table Supplementary Tables 1–9

## Data Availability

Sequence data generated in the LUCAS study have been deposited in the database of the European Genome-phenome Archive (EGA) under accession code EGAS00001005340. Sequence data from the lung validation cohort are available at EGAS00001007248 and for the liver cancer cohort at EGAS00001007249. These data sets are subject to controlled access through EGA owing to restrictions related to sharing of sequence information of study participants. Instructions to download the gnomAD database are available from the gnomAD browser (https://gnomad.broadinstitute.org). ChIP–seq data were downloaded from the ENCODE portal under accession codes ENCFF425LVX, ENCFF098PML and ENCFF574RYG. Somatic mutation calls, tumor purity, coverage statistics as well as mutation signature abundances generated by SigProfiler were downloaded from the International Cancer Genome Consortium Data Portal (https://dcc.icgc.org/releases/PCAWG). Instructions for obtaining access to PCAWG data, including Binary Alignment Map files and germline variant information, are available at https://docs.icgc.org/pcawg/data.

## Code availability

Computer code, software versions and the computing environment for GEMINI are available as a GitHub repository (https://github.com/cancer-genomics/gemini_wflow).
