# Supplementary material for: Single-molecule genome-wide mutation profiles of cell-free DNA for non-invasive detection of cancer
Source: Nat Genet. 2023 Jul 27;55(8):1301–10. doi: 10.1038/s41588-023-01446-3 (PMC10412448; doi:10.1038/s41588-023-01446-3)
Supplement: Supplementary file 1 — Supplementary Note, Supplementary Figs.1–16. [file 41588_2023_1446_MOESM1_ESM.pdf]

nature genetics

Article https://doi.org/10.1038/s41588-023-01446-3

Single-molecule genome-wide mutation profiles of cell-free DNA for non-invasive detection of cancer

In the format provided by the authors and unedited

Supplementary Note

LOD analyses using *in silico* dilutions

We performed two analyses to gain initial insights into the LOD of GEMINI. First, we performed *in silico* dilution of sequence data of samples analyzed from previous cfDNA monitoring studies[11,13] assessed using both targeted mutational analyses as well as GEMINI. In these analyses, whole genome mutational data from cancer patients with known ctDNA levels were diluted with whole genome data from a single non-cancer individual and evaluated for detection using GEMINI. We focused on the three patients who had no radiation to their target lesions, as these could have affected overall mutation levels, using samples more than a week after initiation of targeted therapy. To dilute WGS data from a cancer sample j with an initial median MAF level $\mathrm{MAF}_{\mathrm{j}_{\mathrm{initial}}}$ based on targeted sequencing with a non-cancer sample k to achieve a theoretical median MAF level $\mathrm{MAF}_{\mathrm{final}}$, we first computed the number of equivalents of C:G evaluable bases and C>A sequence changes in the non-cancer sample to mix with the cancer sample as

$$d = \frac{1 - z}{z}$$

where

$$z = \frac{MAF_{final}}{MAF_{j_{initial}}}$$

Next, the diluted number of sequence changes and evaluable bases at bin i, where $i = 1, \ldots, 1144$, was computed by mixing the number of sequence changes and evaluable bases in sample j (y_{ij}^1 and x_{ij}^1 respectively) and sample k (y_{ij}^0 and x_{ij}^0 respectively) as follows

$$y_{ij_{final}}^1 = y_{ij}^1 + d \times \frac{y_{ik}^0}{F_y}$$

and

$$x_{ij_{final}}^1 = x_{ij}^1 + d \times \frac{x_{ik}^0}{F_y}$$

where

$$F_y = \frac{\sum_i x_{ik}^0}{\sum_i x_{ij}^1}$$

Here, F_y accounts for potential differences between k and j with respect to the overall number of evaluable bases. The quantities $y^1_{ij_{final}}$ and $x^1_{ij_{final}}$ were used to compute the GEMINI score using the fixed bins and lung GEMINI model as previously described for values of $\mathrm{MAF_{final}}$ ranging from 0.01% to $\mathrm{MAF_{initial}}$. Sample k used for dilutions (CGPLLU655P) was chosen by randomly selecting one of the two non-cancer samples from the lung cancer validation cohort with a medial lung GEMINI score. In these dilution analyses, we found that at a specificity of 80%, samples tested positive down to median MAFs ranging from 0.09% to 0.29% (median = 0.11%) suggesting a limit of detection of approximately this level for these samples with GEMINI using low coverage whole genome sequencing (Supplementary Fig. 16c). To compare the results to those from analyses of a mutation type not frequently observed in lung cancers, we repeated this procedure using T>G sequence changes and T:A evaluable bases, including generation of a lung GEMINI model as previously described (Methods) using this mutation type and dilution of whole-genome mutational data with the same non-cancer sample (Supplementary Fig. 16d).

As the genome-wide smoking-related mutational burden was unknown for these samples (and may have included individuals not typically screened for lung cancer), we performed a second *in silico* dilution analysis where we combined data from PCAWG samples of lung cancer patients with known mutational landscapes with cfDNA sequence data from a single healthy individual at different concentrations and evaluated the sensitivity of GEMINI for detecting these tumors. Specifically, the regional difference in single molecule C>A frequency at tumor fraction f was computed for the hth PCAWG tumor sample with tumor purity p_h and scaling factor s, reflecting the fold enrichment of ctDNA due to increased evaluable bases in shorter cfDNA fragments due to increased read 1 and 2 overlap, as

$$\text{regional difference}_{hf} = \text{regional difference}_h \times \left(\frac{f}{p_h} \times s\right)$$

For the hth PCAWG tumor sample, we simulated regional differences in single molecule C>A frequencies of 100 non-cancer cfDNA samples at 8x, 4x, 2x, 1x, and 0.5x coverage from a non-cancer cfDNA sample sequenced at high coverage (CGPLLU276P). We computed r_A and r_B in bin sets $\{A_{-h}\}$ and $\{B_{-h}\}$, and derived quantities x^*_{Ah}, x^*_{Bh}, y^*_{Ah} and y^*_{Bh} as previously described (Methods) with the modifications that sampling was performed (i) using CGPLLU276P, and (ii) with replacement. To generate an *in silico* cancer sample with tumor fraction f, $\text{regional difference}_{hf}$ was added to the simulated non-cancer sample with the median regional difference in single molecule C>A frequency at each level of coverage. We determined whether this cancer sample tested positive at 60%, 80%, and 95% specificity based on the 100 non-cancer samples at each coverage. The above procedure was repeated until each tumor sample was determined to be detected or not detected at each coverage across values of f ranging from 0.0001 to 0.1 and assuming $s = 2$ based on previous work[32]. This procedure was repeated for other types of single base changes that are typically less abundant in lung cancers for comparison. To control for potential positive or negative shifts in regional differences in mutation frequencies that are shared across PCAWG samples, for a given mutation type, the median of these values in non-cancer samples was subtracted from that of each cancer sample prior to dilution with non-cancer cfDNA. In these analyses, we found that a majority of individuals with any smoking history

(Supplementary Fig. 16a, n=31) as well as those where the most of mutations were a direct result of smoking exposure (Supplementary Fig. 16b, n=12), were detected at tumor fractions ranging from 0.1%-0.4% at a 2-4x coverage. At higher sequence coverage (8x) and at lower specificities, >75% of individuals could be detected at levels at least 0.04-0.07%.

For the second *in silico* dilution analysis, the tumors that were detected at a tumor fraction of 0.1% (e.g. at 80% specificity and at 1x coverage in Supplementary Fig. 16a) had a median of 42K C>A mutations (range=25K-55K). Repeating the simulation in Fig. 2a using these tumors with only C>A mutations, we found that at a tumor fraction of 0.1% and 1x coverage we expect to observe a median of 32 tumor-derived C>A mutations genome-wide (range 14-52), with a median of 7 tumor-derived mutations in the cancer related bins and 1 in non-cancer related bins. Assuming an estimated two-fold enrichment from increased read 1 / read 2 overlap in shorter fragments of ctDNA, this would lead to a median of ~14 tumor-derived mutations in the cancer related bins and ~2 in non-cancer related bins that could readily be detected using the GEMINI approach.

Overall, the high number of somatic mutations in these tumors combined with the very low background mutation rate achieved by our filters and GEMINI as well as shorter ctDNA fragments was sufficient to detect these tumors at ctDNA levels of 0.1% in our dilution analyses. Detection of tumors with fewer mutations at ctDNA levels of 0.1% would benefit from higher sequencing depth (for example using inexpensive new sequencing approaches[42] and / or advances in reducing errors during library preparation and sequencing). The performance of GEMINI may be even better than these estimates and would benefit from increased read 1 and read 2 (R1/R2) overlap in short fragments known to be enriched in ctDNA. As an example, a previous study showed that physical enrichment of 90-150 bp fragments increased the concentration of ctDNA >2-fold in >95% of cases and >4 fold in >10% of cases[32], and the GEMINI approach would weight these fragments higher than larger fragments due to increased R1/R2 overlap.

Overall, we believe that these LOD levels, especially when considering that the GEMINI method could be combined with DELFI or other genome-wide approaches, could be useful for early detection. Several studies indicate that early stage lung cancers have ctDNA concentrations at similar levels (average of detected stage I MAFs ~0.24%, Supplementary Table 9).

Supplementary Figures

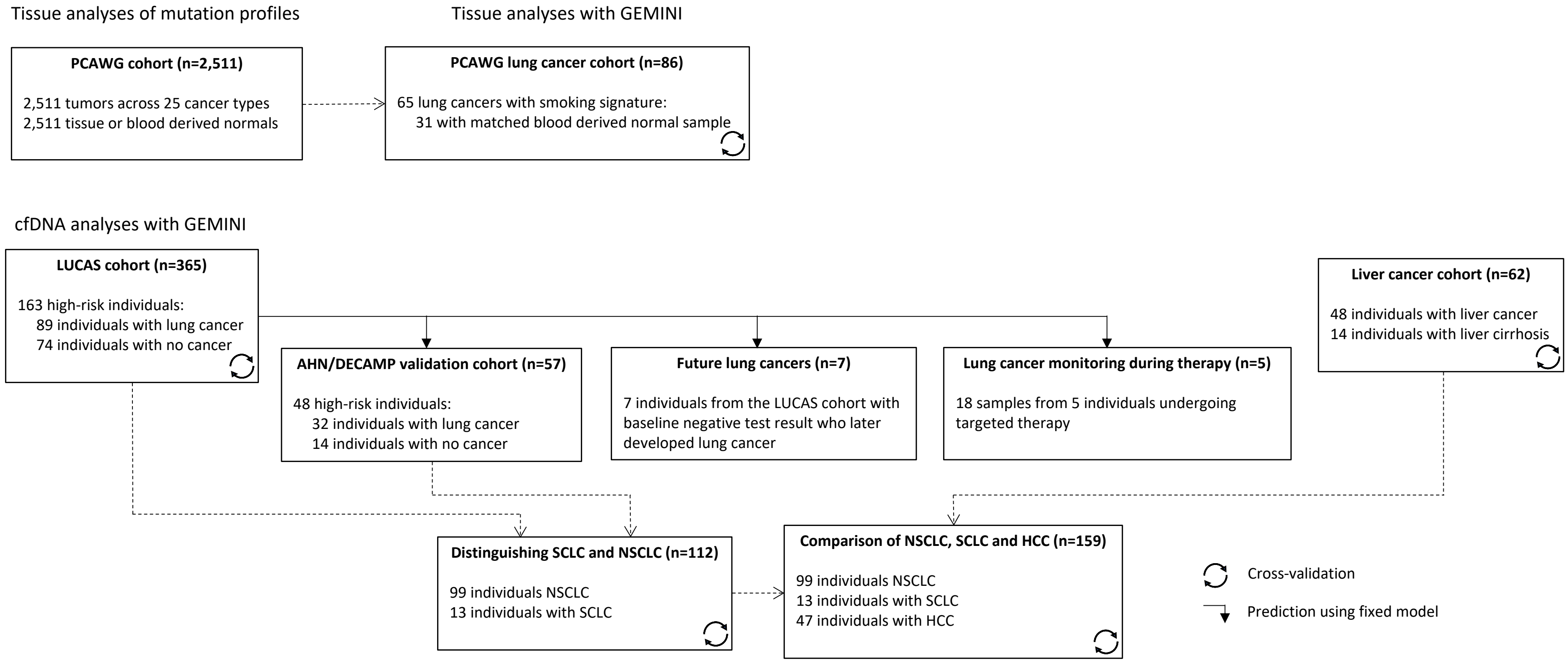

Supplementary Figure 1. Overview of cohorts analyzed. Each box represents a cohort analyzed and indicates whether GEMINI was evaluated with either cross-validation or validated using a fixed model. Dashed lines indicate analyses of cohort subsets for evaluation of individual tumor types or comparison of cancer subtypes. PCAWG, Pan-Cancer Analysis of Whole Genomes; AHN, Allegheny Health Network; DECAMP, Detection of Early Lung Cancer Among Military Personnel; SCLC, small cell lung cancer; NSCLC, non-small cell lung cancer; HCC, hepatocellular carcinoma.

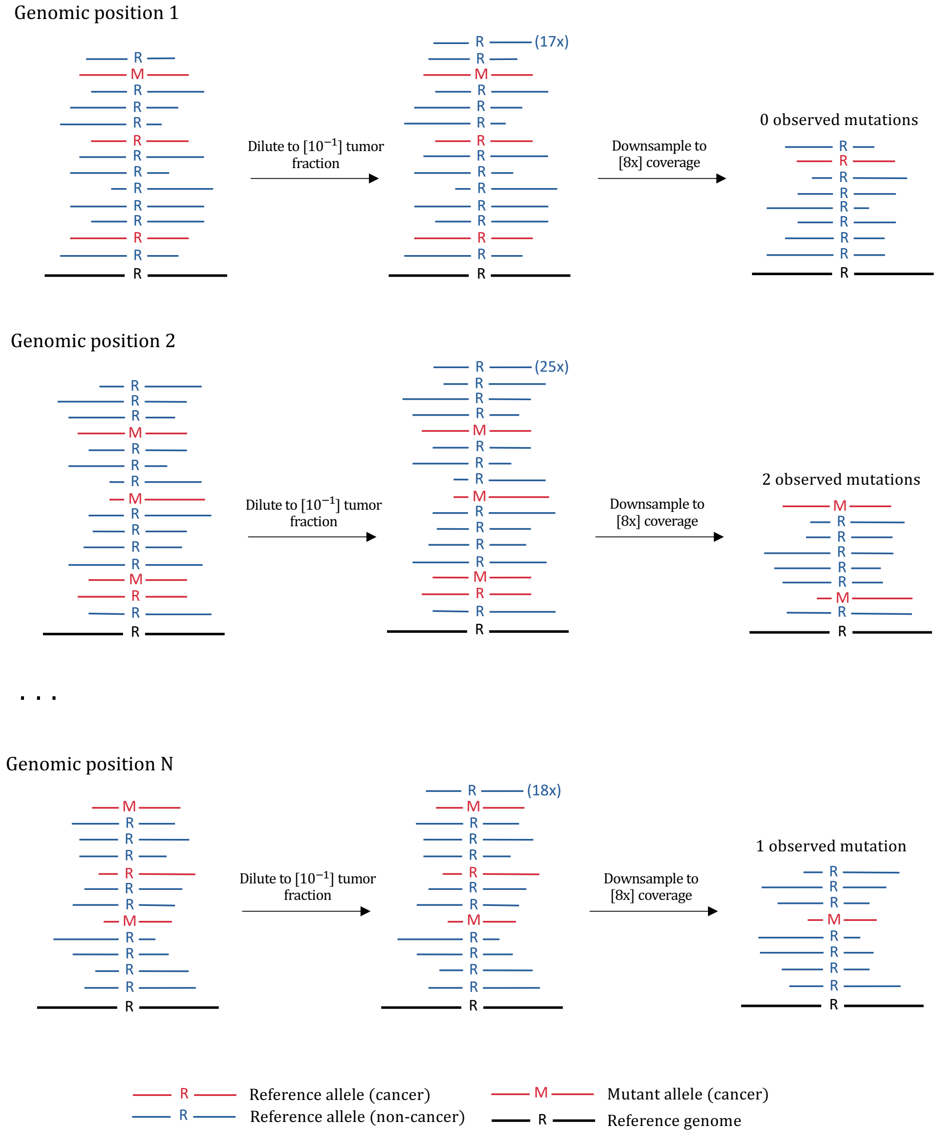

Supplementary Figure 2. Schematic of dilution and downsampling experiment. In this example, we consider a tumor sample that contains N somatic mutations at genomic positions 1, 2, …, N. We begin with 30 non-tumor derived observations and 10 tumor derived mutations (25% tumor purity). During the dilution step, non-tumor observations are spiked in until the desired tumor fraction is achieved. After dilution, fragments are randomly sampled from the set of all fragments to achieve the desired average coverage across genomic positions. The resulting number of observed mutations is counted, and lastly, the proportion of observed mutations that are only observed in a single fragment is computed. In this example, there are 3 observed mutations and one of them is only observed in a single molecule.

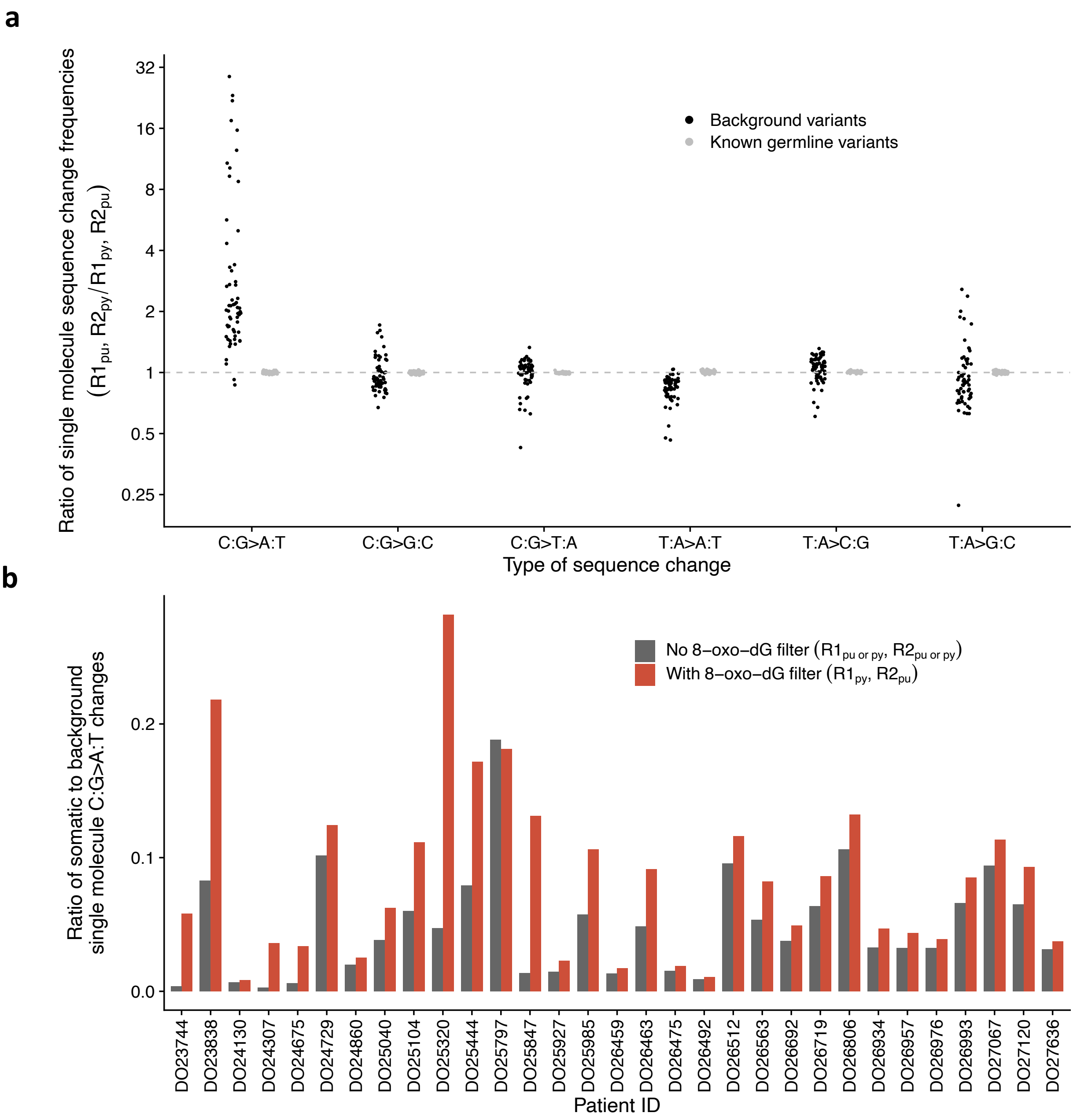

Supplementary Figure 3. Identification of background changes in single molecule sequencing related to 8-oxo-dG damage. a, Ratio of the frequency of each type of single base change in 62 tissue samples from PCAWG (31 lung cancer and 31 blood derived matched normal samples) when prior to mutation purines guanine or adenine (pu) are on read 1 (R1) or pyrimidines cytosine or thymine (py) are on read 2 (R2) to when the pyrimidine is on read 1 and the purine is on read 2 for both background changes and known germline variants. Background changes reflect sequence changes identified through single molecule analyses that were not reported as somatic variants by PCAWG. Here, germline variants reported by PCAWG were also removed from the background variants to enrich for likely artifactual changes. **b,** Ratio of known somatic mutations to background changes identified through single molecule analyses before removing likely 7,8-dihydro-8-oxoguanine (8-oxo-dG) related sequence changes ($R1_{pu\ or\ py}$, $R2_{pu\ or\ py}$), and after filtering these changes where only bases with cytosine on R1 and guanine on read 2 are considered ($R1_{py}$, $R2_{pu}$).

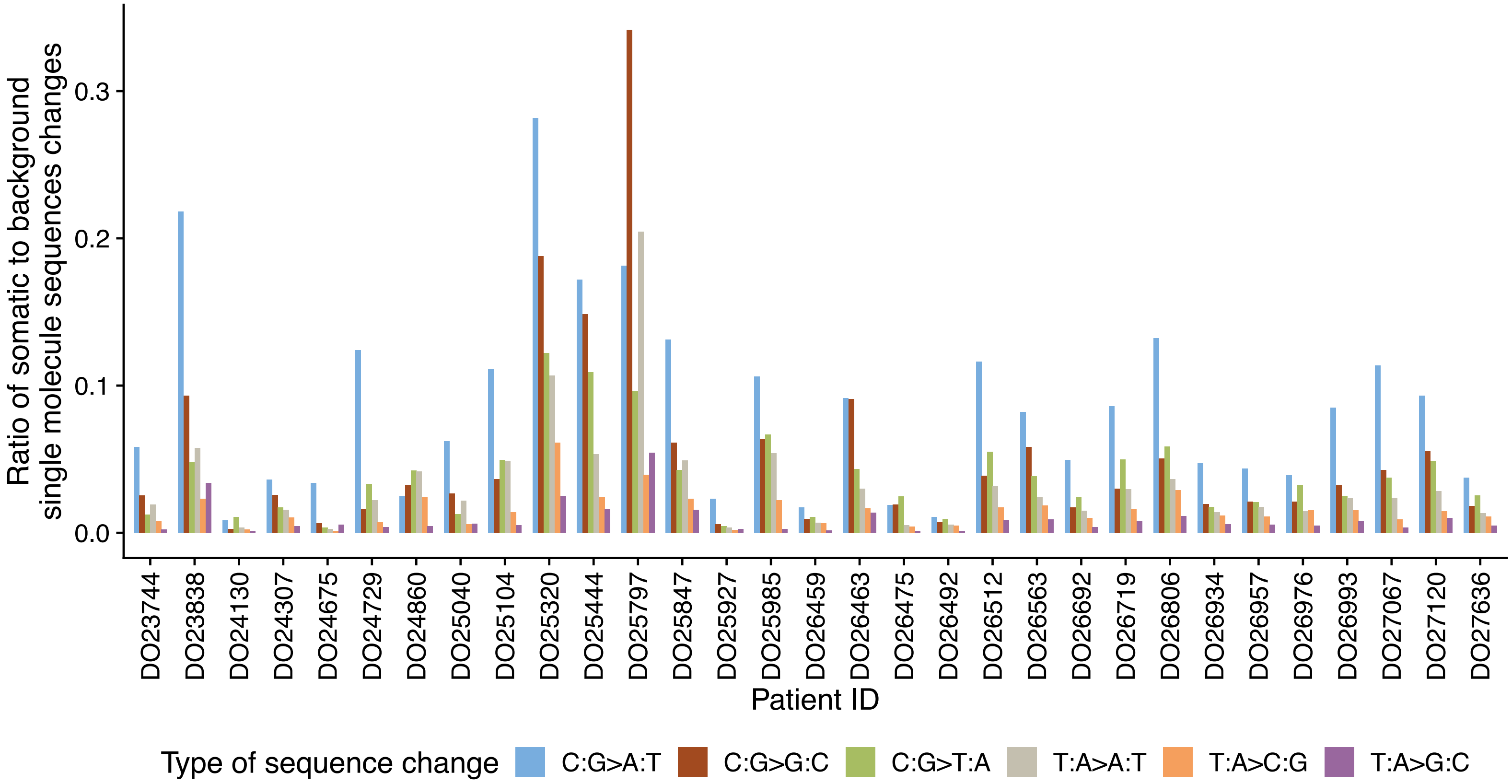

Supplementary Figure 4. Analysis of somatic and background changes across mutation types in PCAWG lung cancers. Ratio of somatic to background changes identified through single molecule analyses after removal of potential 8-oxo-dG related artifacts for each mutation type analyzed. Somatic changes reflect sequence changes identified through single molecule analyses that were also reported as somatic mutations by PCAWG, whereas background changes were identified through single molecule analyses but not reported as somatic mutations by PCAWG. Overall, C:G>A:T changes represented the highest fraction of somatic changes.

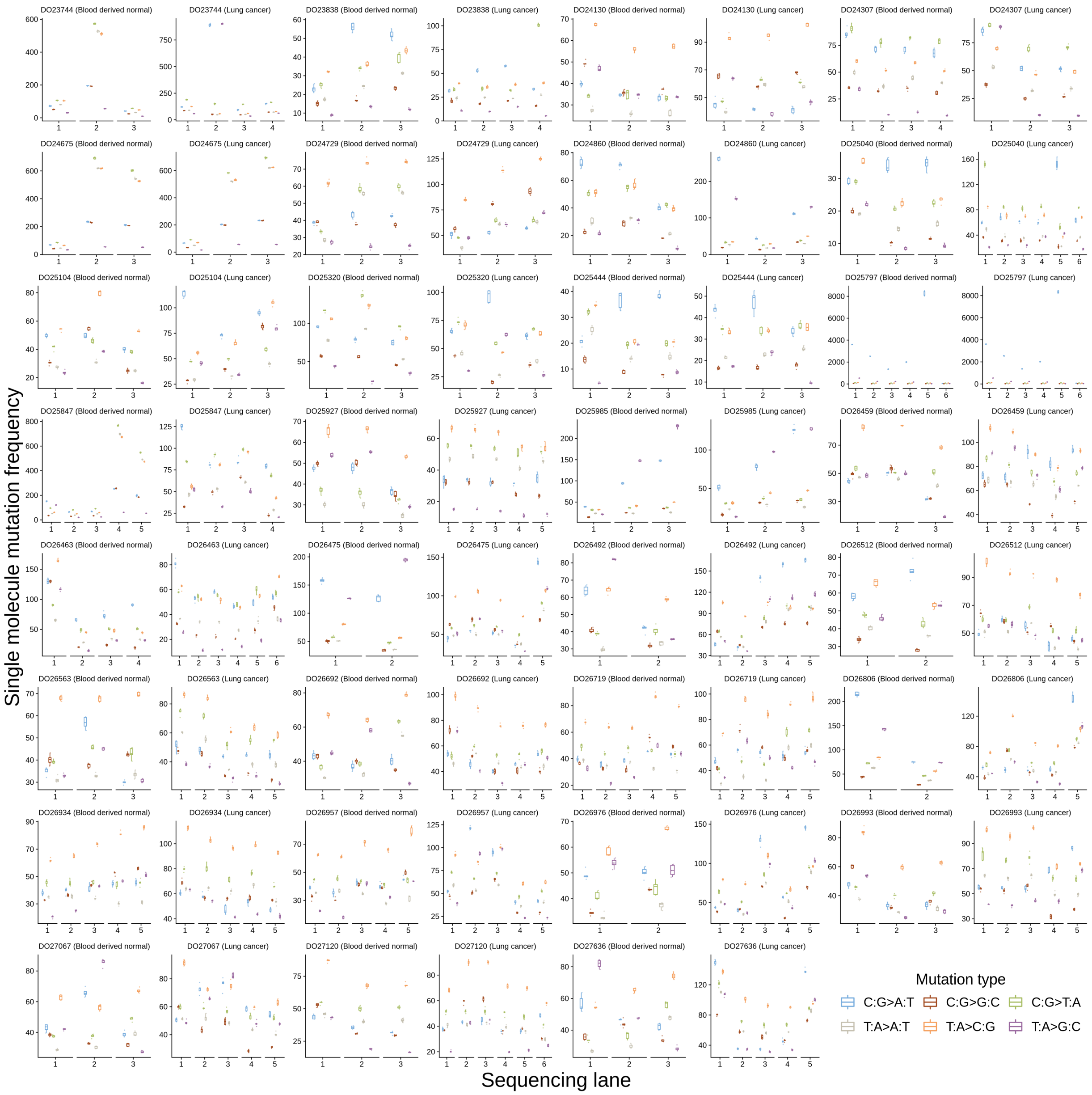

Supplementary Figure 5. Analysis of single molecule sequence changes across sequencing lanes in PCAWG lung cancer and normal samples. Single molecule mutation frequencies in PCAWG lung cancer and blood derived normal samples across sequencing lanes. For each sample, sequencing reads were split into separate Binary Alignment Map (BAM) files based on their associated read group, which indicates the sequencing reads from one lane of an NGS experiment. The resulting BAM files contained a median of 464 million reads (range: 6-738 million). Approximately 1 million reads were randomly sampled 5 times with replacement from each sequencing lane (a maximum of 6 lanes is shown per sample). Single molecule mutation frequencies varied widely within an individual sample depending on the analyzed lane and the type of sequence alteration. All boxplots represent the interquartile range with whiskers drawn to the highest value within the upper and lower fences (upper fence = 0.75 quantile + 1.5 × interquartile range; lower fence = 0.25 quantile – 1.5 × interquartile range). The solid middle line in the boxplot corresponds to the median value. Data points outside the whiskers are shown individually.

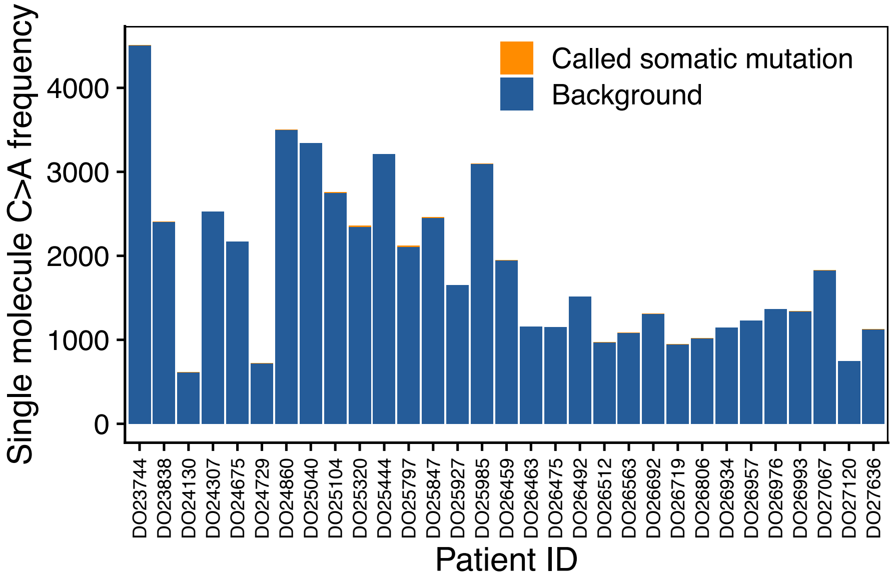

Supplementary Figure 6. Effect of matched WBC filtering in PCAWG lung cancers on enrichment of somatic alterations by single molecule sequencing. Single molecule C>A frequency in PCAWG lung cancers (n=31) after removal of any sequence changes identified in matched blood derived normal samples at >30x coverage. The analysis revealed that subtraction of mutations observed in the matched normal sample was not effective in removing background changes because such alterations typically were observed once and were not present in both tumor and matched non-cancer samples.

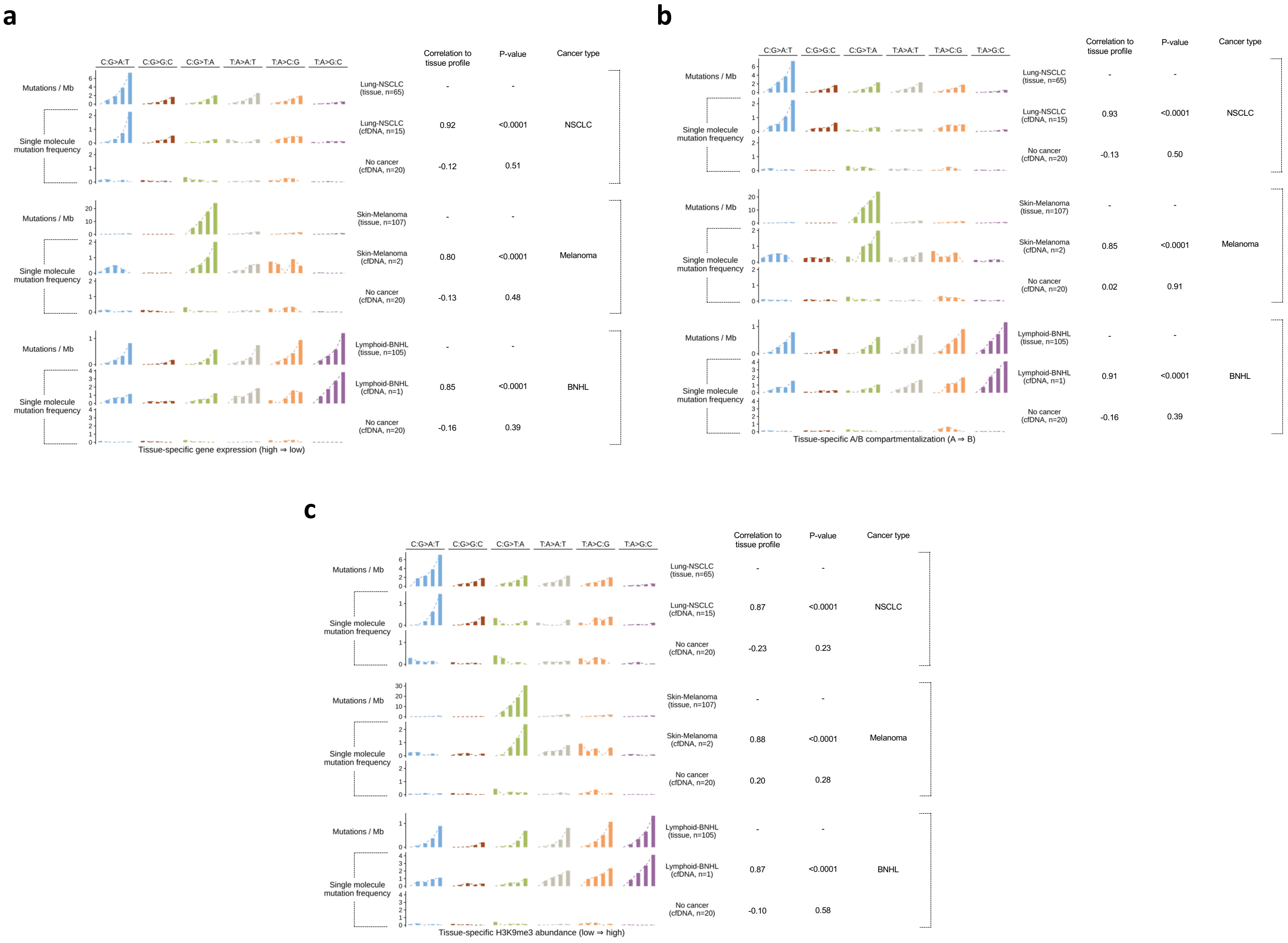

Supplementary Figure 7. Association of single molecule genome-wide mutation profiles of tissue and plasma samples with genomic features. **a-c,** Genome-wide mutation frequencies across strata of tissue-specific gene expression, A/B compartmentalization, and histone 3 lysine 9 tri-

methylation (H3K9me3) abundance, respectively, in tissue and cell-free DNA (cfDNA) from patients with non-small cell lung cancer (NSCLC), melanoma, B-cell non-Hodgkin lymphoma (BNHL), or without cancer. The weighted average of each feature value was computed in 2.5 megabase (Mb) bins, followed by grouping of bins into 5 equal bin sets ordered by feature value. In each bin set, we computed the mutation frequency in tissue at different strata using the number of somatic mutations reported by the PCAWG Consortium per Mb of genome and compared this to the single molecule mutation frequency in plasma using a two-sided Pearson correlation. To account for difference in the overall frequency of each mutation type in each bin in cfDNA, the single molecule mutation frequency in each bin set in a panel of non-cancer samples (n=20) was subtracted from the single molecule mutation frequency in each bin set in cancer and non-cancer cfDNA samples and the resulting values were scaled to have a minimum value of zero for each mutation type and sample type.

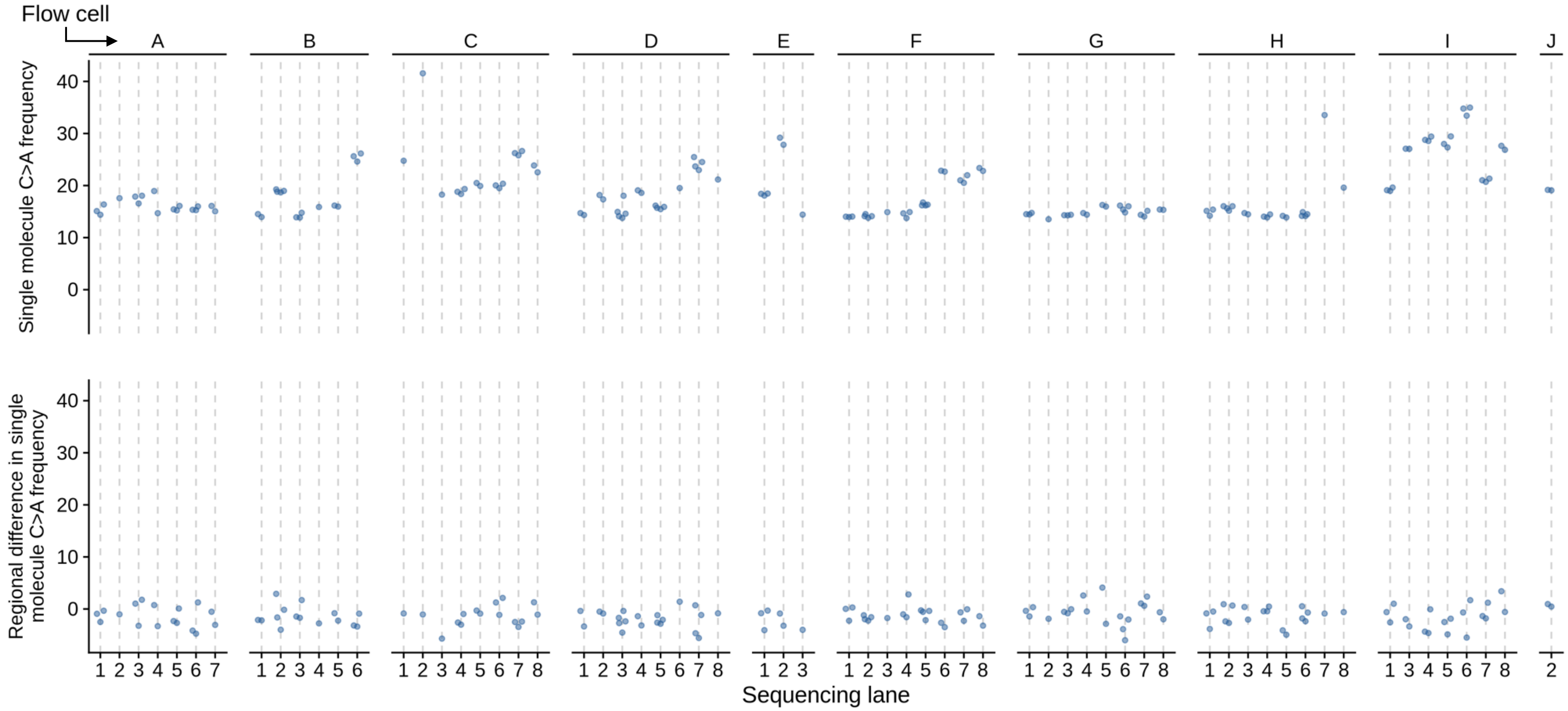

Supplementary Figure 8. Analyses of C>A sequence changes by flow cell and sequencing lane in non-cancer individuals. Single molecule C>A frequencies and regional differences in single molecule C>A frequencies after application of all quality and germline filters across flow cells and sequencing lanes for all non-cancer individuals from the LUCAS cohort (n=158). Although sequencing background mutation rates differed by lane resulting in multiple samples within a sequencing lane having similar single molecule C>A frequencies, explaining 99% of their variance (p<0.0001, F-test, one-sided), this association was eliminated using regional differences in single molecule C>A frequencies obtained with the GEMINI approach (p=0.17, F-test, one-sided). Similar to these analyses, evaluation of all individuals with cancer at baseline (n=199) revealed that sequencing lanes explained 94% of the variance in single molecule C>A frequencies (p<0.0001, F-test, one-sided), and this association was eliminated using regional differences in single molecule C>A frequencies (p=0.44, F-test, one-sided). The sequencing lanes utilized for all samples are reported in Supplementary Table 3.

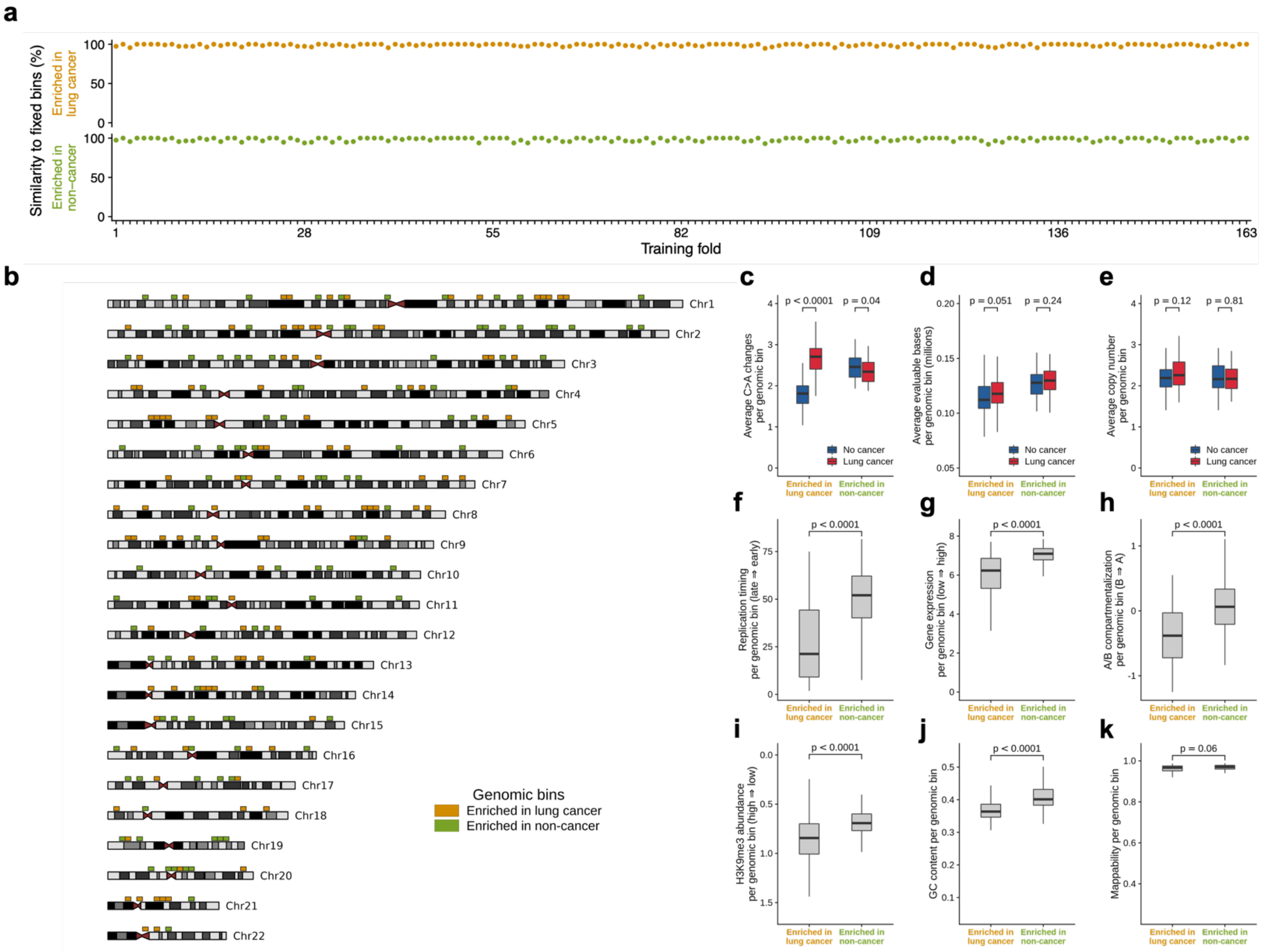

Supplementary Figure 9. Genome-wide fixed bins utilized for analysis of single molecule mutation frequencies and detection of lung cancer in cfDNA. a, Percent similarity of bins identified as being enriched for mutations in lung cancer and non-cancer samples in each training fold compared to the sets of bins utilized in the fixed model that were identified from analyses of all samples. **b,** Chromosomal location of bins

enriched in mutations in cfDNA of patients with lung cancer and individuals without cancer. The approximate location of Giemsa-stained chromosome bands are shown in grayscale and centromeres are shown in red. **c,** Compared to individuals without cancer (n=74), samples from those with lung cancer (n=89) had more C>A changes per genomic bin across samples in bins enriched in lung cancer ($p<0.0001$, Wilcoxon rank sum test, two-sided) and fewer of these changes in bins enriched in non-cancer ($p=0.04$, Wilcoxon rank sum test, two-sided). **d-e,** The average number of evaluable bases and copy number per genomic bin were similar in individuals with (n=89) and without (n=74) lung cancer in each bin set ($p>0.05$ for each comparison, Wilcoxon rank sum test, two-sided). Copy number was estimated using ichorCNA. **f-k,** Bins in the fixed model were associated with replication timing from IMR90 cells (**f**), gene expression from TCGA lung cancers represented as $\log_{10}$(TPM) (**g**), A/B compartmentalization from TCGA lung cancers (**h**), and histone 3 lysine 9 tri-methylation (H3K9me3) abundance from ChIP-seq of A549 cells (**i**), GC (guanine-cytosine) content obtained from the hg19 reference genome (**j**), ($p<0.0001$ for each comparison, Wilcoxon rank sum test, two-sided) but not sequence mappability (**k**) ($p=0.06$, Wilcoxon rank sum test, two-sided) (n=114 bins in each bin set). Boxplots represent the interquartile range with whiskers drawn to the highest value within the upper and lower fences (upper fence = 0.75 quantile + 1.5 $\times$ interquartile range; lower fence = 0.25 quantile – 1.5 $\times$ interquartile range). The solid middle line in the boxplot corresponds to the median value. Chr, Chromosome.

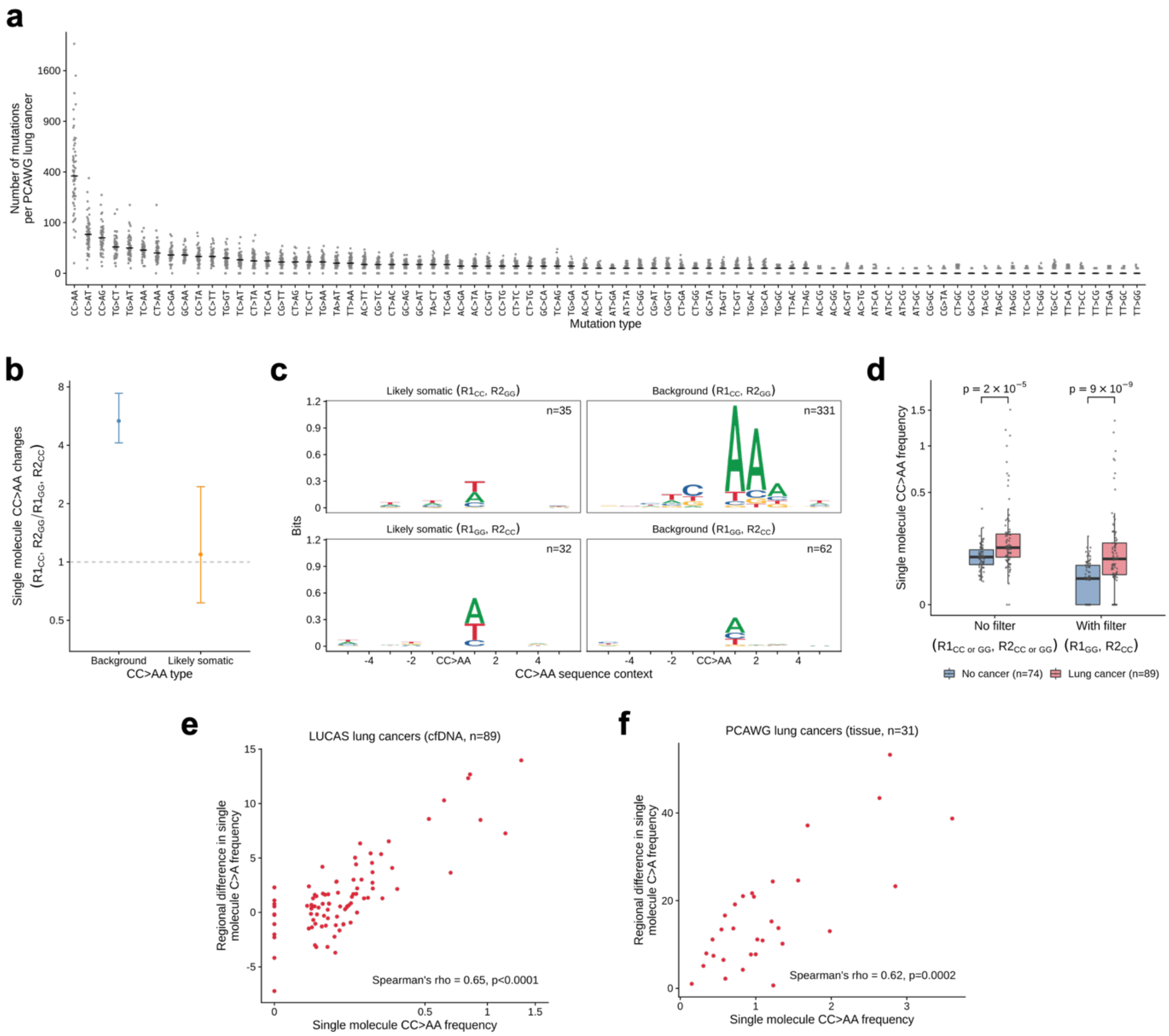

Supplementary Figure 10. Analyses of doublet base substitutions. a, Number of somatic doublet base substitutions in Pan-Cancer Analysis of Whole Genomes (PCAWG) lung cancers from smoking individuals (n=65) with the median value indicated. **b,** Ratio of single molecule CC>AA frequencies with CC or CC>AA in read 1 and GG or GG>TT in read 2 ($R1_{CC}$, $R2_{GG}$) relative to when GG or GG>TT is in read 1 and CC or CC>AA is in read 2 ($R1_{GG}$, $R2_{CC}$) aggregated across the high-risk LUCAS cohort. Here, background CC>AA changes represent alterations observed once in non-cancer individuals, whereas likely somatic changes represent those that were private to a lung cancer patient and observed in >1 fragment. There were 67 private CC>AA changes in >1 fragment across 89 individuals with lung cancer, and only one across 74 non-cancer individuals, indicating that most of these alterations were likely somatic in origin. Vertical bars represent 95% bootstrap confidence intervals for the ratios (indicated by dots). Only Background CC>AA changes were preferentially detected as $R1_{CC}$, $R2_{GG}$. **c,** Sequence context surrounding CC>AA changes (+/-5bp) in the high-risk LUCAS cohort with the number of mutations indicated. The height at each position indicates information content measured in bits. Background changes detected as $R1_{CC}$, $R2_{GG}$ preferentially occur upstream of 'A' bases. **d,** CC>AA frequencies in the high-risk LUCAS cohort were elevated in individuals with lung cancer with a larger separation after filtering $R1_{GG}$, $R2_{CC}$ changes ($p=2 \times 10^{-5}$ before and $p=9 \times 10^{-9}$ after filter, Wilcoxon rank sum test, two-sided). **e-f,** CC>AA frequencies were positively correlated with regional differences in C>A frequencies in cell-free (cfDNA) (Spearman's correlation coefficient = 0.65, p<0.0001, two-sided) (**e**) and tissue (Spearman's correlation coefficient = 0.62, p<0.0001, two-sided) (**f**) after filtering $R1_{CC}$, $R2_{GG}$ changes. All boxplots represent the interquartile range with whiskers drawn to the highest value within the upper and lower fences (upper fence = 0.75 quantile + 1.5 × interquartile range; lower fence = 0.25 quantile – 1.5 × interquartile range). The solid middle line in the boxplot corresponds to the median value.

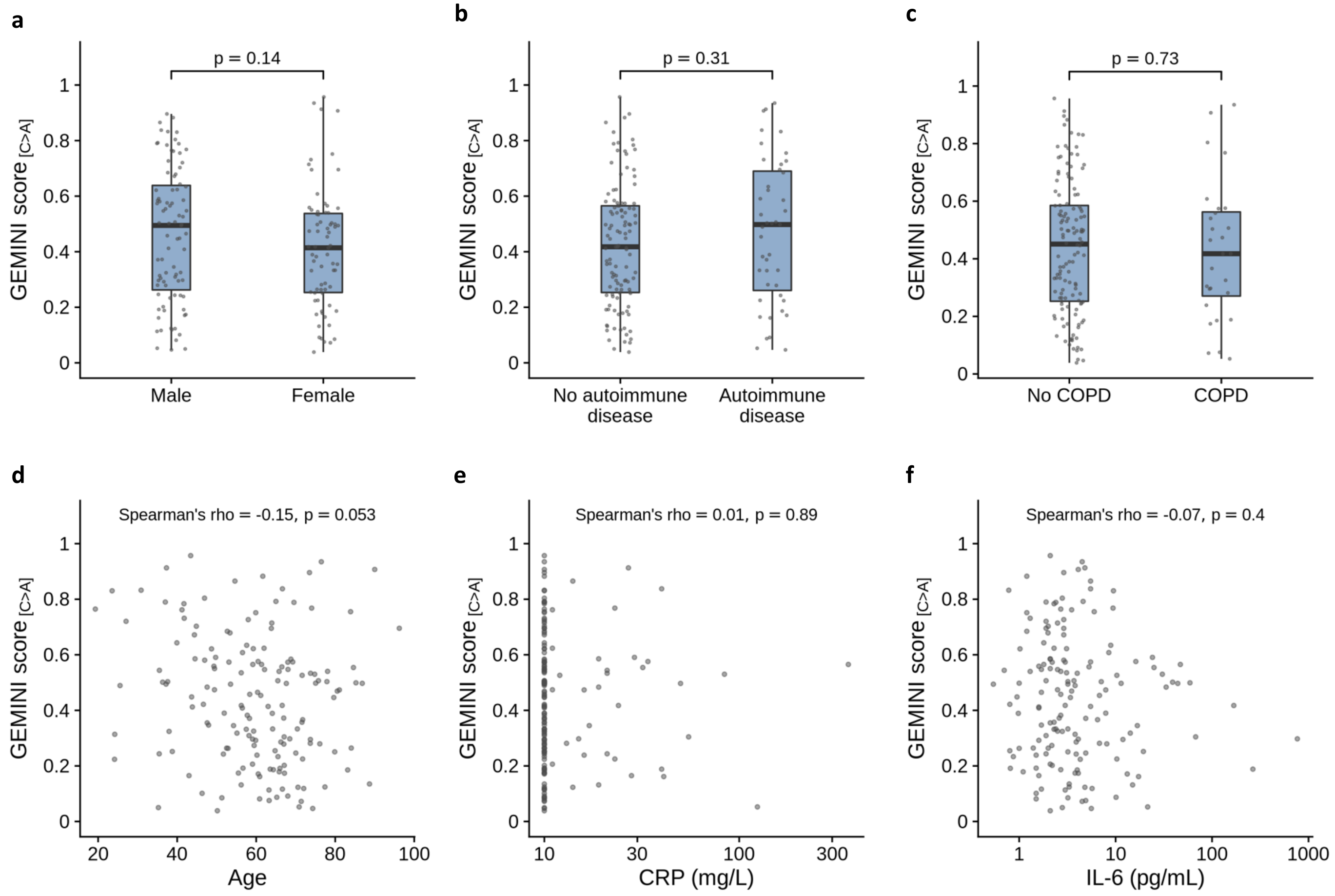

Supplementary Figure 11. Effect of clinical characteristics on GEMINI scores in non-cancer individuals in the LUCAS cohort. GEMINI scores are shown for **a,** males (n=87) and females (n=71), **b,** individuals with (n=43) or without (n=115) autoimmune disease, **c,** individuals with (n=28) or without (n=130) chronic obstructive pulmonary disease (COPD), **d,** individuals of different ages, and **e-f**, compared to C-reactive protein (CRP) measured in milligrams per liter (mg/L), and Interleukin 6 (IL-6) levels measured in picograms per milliliter (pg/mL). P-values were derived from two-sided Wilcoxon rank sum tests (**a-c**) and two-sided Spearman's correlations (**d-f**)**.** All boxplots represent the interquartile range with whiskers drawn to the highest value within the upper and lower fences (upper fence = 0.75 quantile + 1.5 × interquartile range; lower fence = 0.25 quantile – 1.5 × interquartile range). The solid middle line in the boxplot corresponds to the median value.

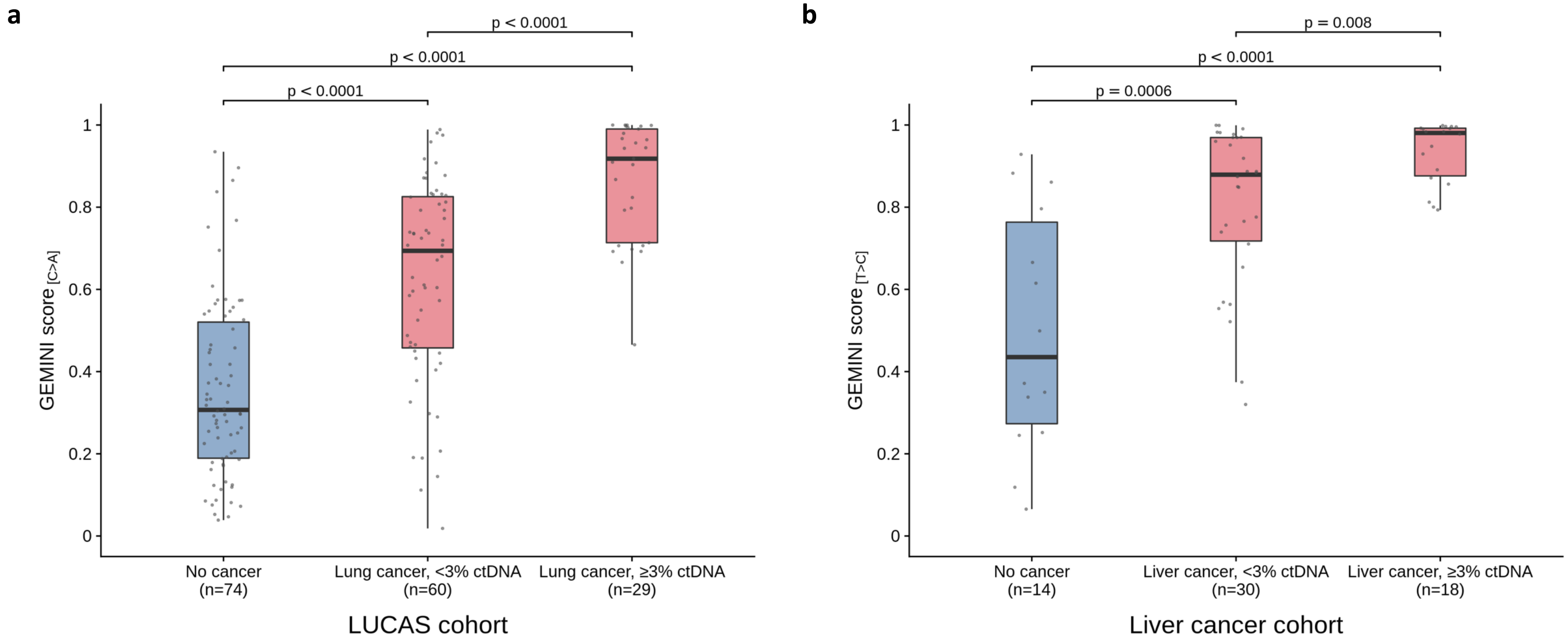

Supplementary Figure 12. GEMINI scores reflect tumor DNA content in cfDNA. a, GEMINI scores in the high-risk LUCAS cohort in individuals without cancer and individuals with lung cancer at different levels of circulating tumor DNA (ctDNA). A score >0.55 reflects a positive test for detection of lung cancer at 80% specificity. P-values were obtained from two-sided Wilcoxon rank sum tests. **b,** GEMINI scores in the liver cancer cohort in individuals with cirrhosis and individuals with liver cancer that have <3% or ≥3% ctDNA. A score >0.86 reflects a positive test for detection of liver cancer at 80% specificity. The percentage of ctDNA in each sample was estimated using ichorCNA. P-values were obtained from two-sided Wilcoxon rank sum tests. All boxplots represent the interquartile range with whiskers drawn to the highest value within the upper and lower fences (upper fence = 0.75 quantile + 1.5 × interquartile range; lower fence = 0.25 quantile – 1.5 × interquartile range). The solid middle line in the boxplot corresponds to the median value.

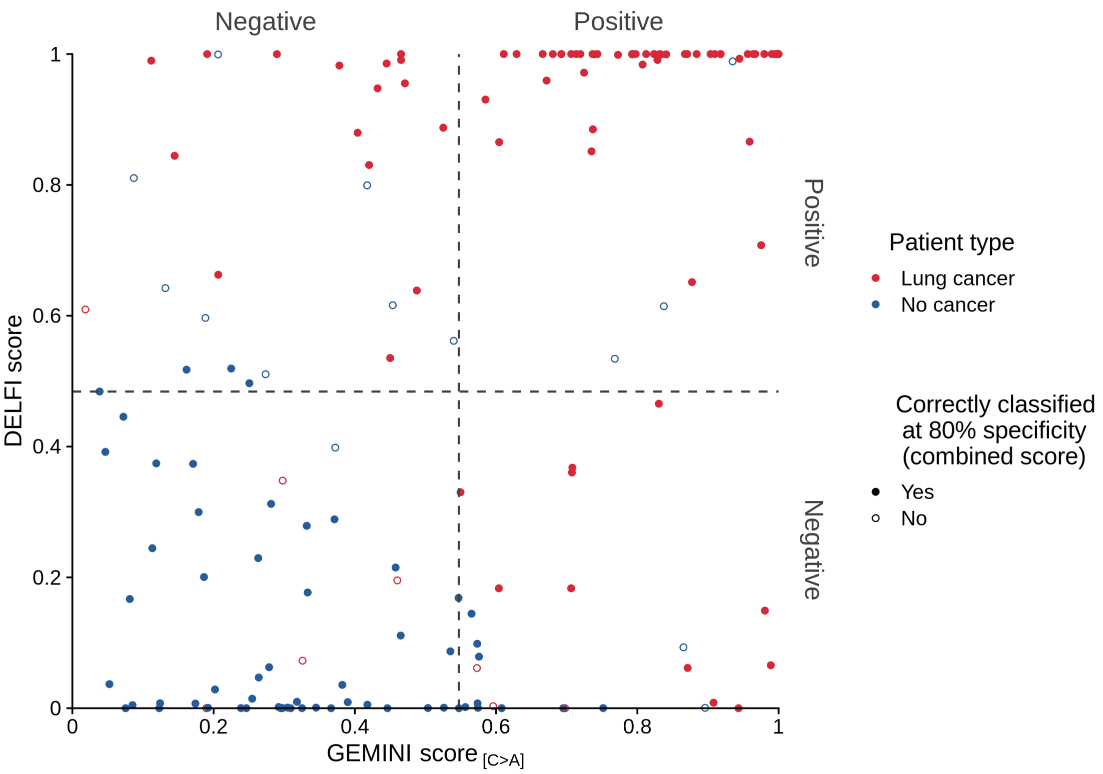

Supplementary Figure 13. GEMINI and DELFI scores as well as their combined performance for detecting cancer in the LUCAS cohort. GEMINI and DELFI scores are shown for each patient in the high-risk LUCAS cohort (n=163). Vertical and horizontal dashed lines indicate the threshold for a positive GEMINI and DELFI test respectively at 80% specificity, while filled circles indicate a positive test by the combined approach at the same specificity. Several individuals with cancer are detected by one approach but not the other, and the combined score detected more individuals with lung cancer compared to either approach in isolation.

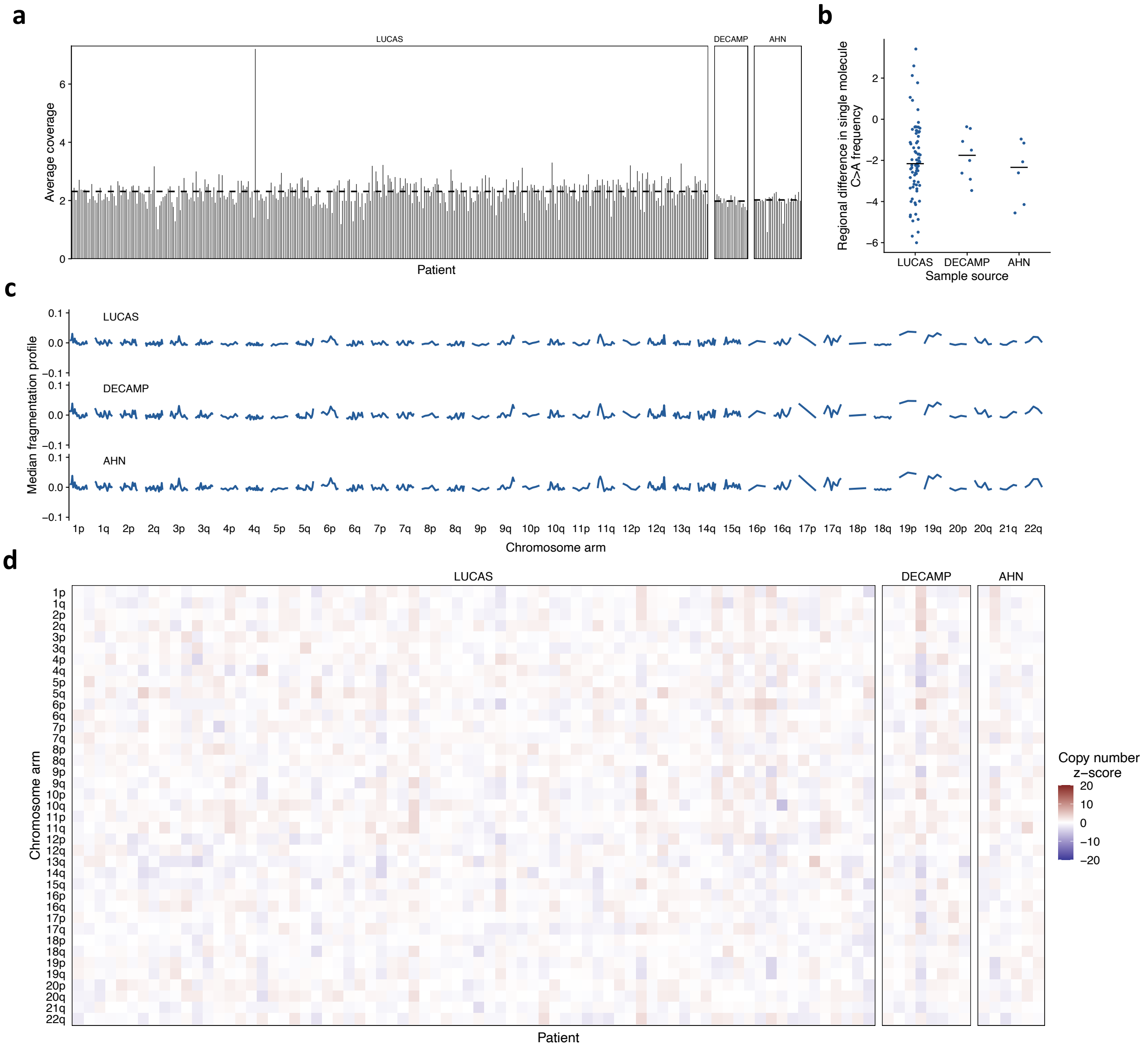

Supplementary Figure 14. Comparison of cfDNA characteristics across non-cancer patients in LUCAS, DECAMP, and AHN cohorts. a, Average genome-wide coverage in non-cancer samples across cohorts. The horizontal dashed lines represent the median coverage of samples in each cohort. **b,** Regional differences in single molecule C>A frequencies are similar between cohorts (p=0.17, Kruskal-Wallis test) (n: LUCAS = 74, Detection of Early Lung Cancer Among Military Personnel (DECAMP) = 8, Allegheny Health Network (AHN) = 6). Solid horizontal lines represent the median in each group. **c,** For each non-cancer sample, the ratio of short (100-150bp) to long (151-220bp) fragments was computed in 473 non-overlapping 5Mb bins and mean-centered. Median fragmentation profiles represent the median of these values across samples in each bin and are highly correlated between cohorts (Pearson correlation coefficient >0.97 for each pairwise comparison). **d,** Chromosomal arm-level Z-scores in non-cancer samples are similar between cohorts (p>0.05 for each chromosomal arm, Kruskal-Wallis test with Bonferroni correction).

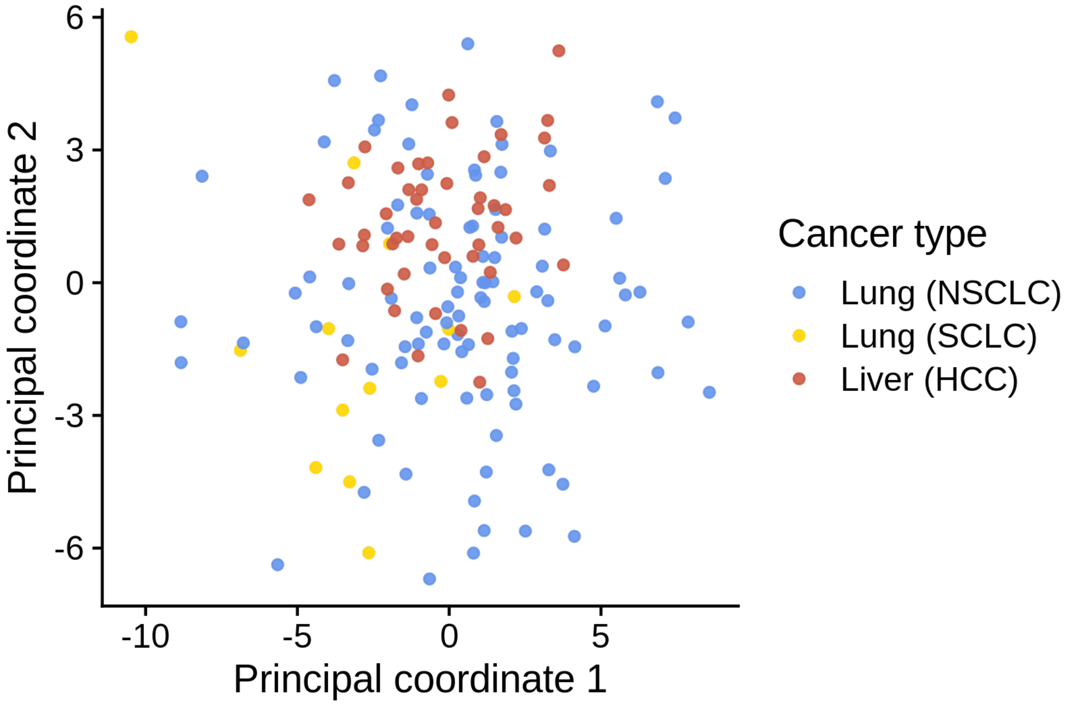

Supplementary Figure 15. Principal coordinate analysis in patients with cancer after excluding the most frequent mutation types. The regional difference in single molecule mutation frequency was computed between non-small cell lung cancer (NSCLC), small cell lung cancer (SCLC), and hepatocellular carcinoma (HCC) using a leave-one-out procedure for C>G, C>T, T>A and T>G mutations, yielding 12 feature values. A Euclidean distance matrix reflecting pairwise differences between samples was generated from these 12 feature values. A principal coordinate analysis of the Euclidean distance matrix revealed a reduced separation of samples by cancer type compared to when C>A and T>C mutations were also analyzed (**Fig. 5f**).

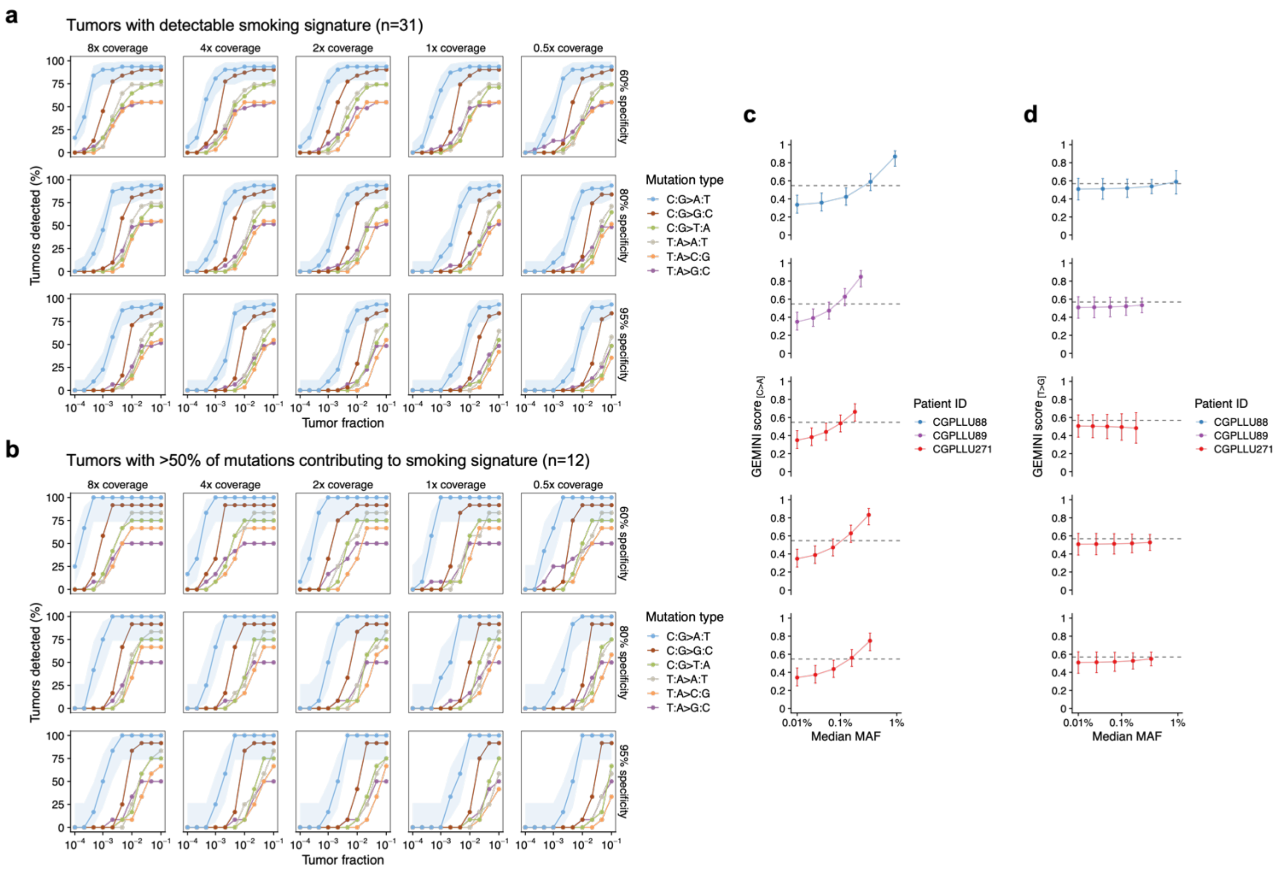

Supplementary Figure 16. *In silico* evaluation of the limit of detection of GEMINI. a-b, Proportion of tumors with known mutational landscapes detected using analyses of each type of single base change across a range of tumor fractions from *in silico* dilution analyses where data from PCAWG samples of lung cancer patients with detectable smoking related Signature 4 (**a**) or with >50% of mutations contributing to Signature 4 (**b**), was combined with cfDNA sequence data from a healthy individual (Methods). The shaded regions reflect 95% confidence intervals for proportions (indicated by dots) based on a binomial model for C:G>A:T sequence changes. **c-d,** GEMINI scores with 95% confidence intervals derived from analyses of C>A changes (**c**) as well as T>G changes that were relatively infrequent in lung cancers (**d**), at different mutant allele fraction (MAF) levels from *in silico* dilution of sequence data of cancer samples from the lung cancer monitoring study that were assessed using both targeted mutational analyses as well as GEMINI with sequence data from a healthy individual (Methods). The samples analyzed using *in silico* dilutions included one sample from each of patients CGPLLU88 and CGPLLU89 and three samples from patient CGPLLU271. The highest median MAF in each figure reflects the initial median MAF prior to dilution. The horizontal dashed line indicates the threshold for detection of lung cancer by GEMINI at 80% specificity.
