# Supplementary material for: Single-molecule genome-wide mutation profiles of cell-free DNA for non-invasive detection of cancer
Source: Nat Genet. 2023 Jul 27;55(8):1301–10. doi: 10.1038/s41588-023-01446-3 (PMC10412448; doi:10.1038/s41588-023-01446-3)
Supplement: Supplementary file 2 — Reporting Summary [file 41588_2023_1446_MOESM2_ESM.pdf]

nature portfolio

Corresponding author(s): Velculescu V.E., Scharpf R.B.

Last updated by author(s): Jun 14, 2023

Reporting Summary

Nature Portfolio wishes to improve the reproducibility of the work that we publish. This form provides structure for consistency and transparency in reporting. For further information on Nature Portfolio policies, see our Editorial Policies and the Editorial Policy Checklist.

Statistics

For all statistical analyses, confirm that the following items are present in the figure legend, table legend, main text, or Methods section.

n/a	Confirmed	
☐	☒	The exact sample size (*n*) for each experimental group/condition, given as a discrete number and unit of measurement
☐	☒	A statement on whether measurements were taken from distinct samples or whether the same sample was measured repeatedly
☐	☒	The statistical test(s) used AND whether they are one- or two-sided<br>*Only common tests should be described solely by name; describe more complex techniques in the Methods section.*
☐	☒	A description of all covariates tested
☐	☒	A description of any assumptions or corrections, such as tests of normality and adjustment for multiple comparisons
☐	☒	A full description of the statistical parameters including central tendency (e.g. means) or other basic estimates (e.g. regression coefficient) AND variation (e.g. standard deviation) or associated estimates of uncertainty (e.g. confidence intervals)
☐	☒	For null hypothesis testing, the test statistic (e.g. *F*, *t*, *r*) with confidence intervals, effect sizes, degrees of freedom and *P* value noted<br>*Give P values as exact values whenever suitable.*
☒	☐	For Bayesian analysis, information on the choice of priors and Markov chain Monte Carlo settings
☐	☒	For hierarchical and complex designs, identification of the appropriate level for tests and full reporting of outcomes
☐	☒	Estimates of effect sizes (e.g. Cohen's *d*, Pearson's *r*), indicating how they were calculated

Our web collection on statistics for biologists contains articles on many of the points above.

Software and code

Policy information about availability of computer code

Data collection	N/A
Data analysis	For processing unaligned reads, we used fastp (>=0.20.0), Bowtie2 (2.3.5.1), SAMtools (>=1.9), Sambamba (>=0.7.1), and pysam (0.16.0.1). Other software for data analysis include recount3 (1.0.2), and ichorCNA (0.3.2). Custom scripts for GEMINI were implemented in R (>= 3.6.1) and Python (3.8.2) and are maintained in a GitHub repository (https://github.com/cancer-genomics/gemini_wflow) and will be made available upon publication. All R packages and dependencies from these customized scripts were captured programmatically and made available in the GitHub repository.

For manuscripts utilizing custom algorithms or software that are central to the research but not yet described in published literature, software must be made available to editors and reviewers. We strongly encourage code deposition in a community repository (e.g. GitHub). See the Nature Portfolio guidelines for submitting code & software for further information.

Data

Policy information about availability of data

All manuscripts must include a data availability statement. This statement should provide the following information, where applicable:

- Accession codes, unique identifiers, or web links for publicly available datasets
- A description of any restrictions on data availability
- For clinical datasets or third party data, please ensure that the statement adheres to our policy

Sequence data generated in the LUCAS study have been deposited at the database of European Genome-Phenome Archive (EGA) under accession code EGAS00001005340. Sequence data from the lung validation cohort are available at EGAS00001007248 and for the liver cancer cohort at EGAS00001007249. These datasets are subject to controlled access through EGA due to restrictions related to sharing of sequence information of study participants. Instructions to download

the gnomAD database are available from the gnomAD browser (https://gnomad.broadinstitute.org/). ChIp-seq data was downloaded from the ENCODE portal under accession codes ENCFF425LVX, ENCFF098PML, and ENCFF574RYG. Somatic mutation calls, tumor purity, coverage statistics, as well as mutation signature abundances generated by SigProfiler were downloaded from the International Cancer Genome Consortium (ICGC) Data Portal (https://dcc.icgc.org/releases/PCAWG). Instructions for obtaining access to PCAWG data, including BAM files and germline variant information are available at https://docs.icgc.org/pcawg/data/.

Field-specific reporting

Please select the one below that is the best fit for your research. If you are not sure, read the appropriate sections before making your selection.

☒ Life sciences ☐ Behavioural & social sciences ☐ Ecological, evolutionary & environmental sciences

For a reference copy of the document with all sections, see nature.com/documents/nr-reporting-summary-flat.pdf

Life sciences study design

All studies must disclose on these points even when the disclosure is negative.

Sample size

The prevalence of undiagnosed cancer cases in the prospectively collected LUCAS cohort was expected to be high (approximately 30 cancers per 100 non-cancer individuals). Assuming a noninvasive test for cancer with a specificity of 0.85 in a study of nearly 400 participants, our study would provide an estimate of sensitivity with a margin of error of 0.05 or smaller. In other cases, sample sizes were chosen based on sample availability.

Data exclusions

3 subjects were excluded from the LUCAS cohort due to failure in the sequencing step and no data were able to be retrieved from the sequencer, therefore no data were available for analysis. The above is a pre-established requirement for inclusion of samples in the the cohorts analyzed.

A previously published lung cancer cohort (Mathios et al., Nature Commun, 2021) was not used in this study as it included samples from sources that did not collect information related to smoking exposure. As GEMINI scores were significantly correlated with smoking history in individuals with and without cancer, a validation cohort likely containing individuals without a smoking history would be inappropriate as cfDNA mutation profiles and GEMINI scores of samples from these individuals would not be expected to reflect those of individuals in the high-risk LUCAS cohort (smoking history >=20 pack years) used for model training.

Replication

We have shown successful use of the method in 8 different settings: i) PCAWG lung cancers, ii) high-risk LUCAS cohort, iii) AHN/DECAMP validation lung cancer cohort, iv) lung cancers detected after baseline blood draw in LUCAS, v) monitoring lung cancer patients treated with targeted therapy, vi) liver cancer cohort, and vii) comparison of regional mutation profiles among lung cancer subtypes and liver cancers, as well as viii) distinguishing NSCLC from SCLC. Three of these cohorts (iii, iv, v) used a locked machine learning model.

Randomization

Cancer and non-cancer individuals were prospectively collected in the LUCAS cohort. Cancer and non-cancer individuals were randomly allocated to cfDNA extraction batches and library preparation batches. Due to the prospective study design and the random allocation of cancer and non-cancer samples to each batch, we expect that both known and unknown confounders would be similar across the collection of batches.

Blinding

The investigators were not blinded to diagnosis for the LUCAS or liver cancer cohorts since these cohorts was used for training of machine learning models. While the investigators that prepared the libraries for the AHN/DECAMP validation cohort had to know the diagnosis of each individual used to randomize the samples across library preparation, these individuals were not involved in the computational aspects of this project including machine learning. For other analyses, investigators were not blinded to group allocation as knowledge of group labels was necessary for stastistical testing.

Reporting for specific materials, systems and methods

We require information from authors about some types of materials, experimental systems and methods used in many studies. Here, indicate whether each material, system or method listed is relevant to your study. If you are not sure if a list item applies to your research, read the appropriate section before selecting a response.

Materials & experimental systems

n/a	Involved in the study	
☒	☐	Antibodies
☒	☐	Eukaryotic cell lines
☒	☐	Palaeontology and archaeology
☒	☐	Animals and other organisms
☐	☒	Human research participants
☒	☐	Clinical data
☒	☐	Dual use research of concern

Methods

n/a	Involved in the study	
☒	☐	ChIP-seq
☒	☐	Flow cytometry
☒	☐	MRI-based neuroimaging

Human research participants

Policy information about studies involving human research participants

Population characteristics	Tissue samples in the PCAWG Consortium consisted of lung cancer and matched solid tissue or blood cells from 2,511 donors. The PCAWG lung cancer cohort consisted of 30 females and 56 males who were diagnosed with lung cancer between ages 41 and 83. Among these individuals, 38 had lung adenocarcinoma and 48 had lung squamous cell carcinoma.<br><br>The LUCAS cohort consisted of patients of age 19-96. There were 186 male patients and 179 female patients. There were 158 patients with no prior, baseline, or future cancers, 114 patients with baseline lung cancer, 15 patients with a lung metastasis, and 78 patients without lung cancer at the time of blood collection, but with either earlier or later lung cancers or another cancer type. Treatment for the patients with lung cancer in the LUCAS cohort was performed in accordance with the most updated ESMO guidelines at the time of treatment.<br><br>The AHN/DECAMP validation cohort consisted of 57 patients of age 47-86. There were 31 male patients and 26 female patients. There were 42 patients with lung cancer and 15 patients with a benign lung nodule.<br><br>The liver cancer cohort consisted of 62 patients of age 32-81. There were 13 female and 49 male patients. There were 48 patients with liver cancer and 14 patients with cirrhosis.<br><br>The lung cancer monitoring cohort consisted of 18 samples from 5 patients undergoing therapy with tyrosine kinase inhibitors. Patients were between the ages of 50 and 73 and were 60% female.
Recruitment	Patients involved in the PCAWG Consortium were recruited by the participating centres following local protocols.<br><br>The LUCAS cohort represents a prospectively collected group of patients age >18 that presented in the Department of Respiratory Medicine, Infiltrate Unite, Bispebjerg Hospital, Copenhagen with a positive imaging finding on a chest X-ray or a chest CT. Patients with known cancer and active disease or who were undergoing treatment at the time of enrollment were excluded. The collection lasted from September 2012 to March of 2013. The LUCAS cohort consists of patients with predominantly symptomatic disease, although most symptoms are common symptoms that the majority of patients in a screening program would have (such as cough, sputum production, dyspnea).<br><br>For the AHN/DECAMP validation cohort, individuals were enrolled either through the Detection of Early Lung Cancer Among Military Personnel (DECAMP) Consortium, or through screening efforts at the Allegheny Health Network (AHN). The DECAMP-1 protocol included current or former cigarette smokers with ≥20 pack-year exposure and radiological findings indicating an indeterminate pulmonary nodule of 0.7 to 3.0cm in size identified within 12 months prior to enrollment with an additional CT scan within 3 months prior to enrollment. Individuals enrolled at the AHN were identified based on eligibility for high-risk screening for lung cancer using low-dose helical CT scanning or an indication for lung cancer screening based on other high-risk characteristics such as family history of lung cancer.<br><br>Patients with lung cancer undergoing treatment with tyrosine kinase inhibitors at University of California San Diego (San Diego, CA) or Johns Hopkins University (Baltimore, MD) were included in our study. The study population included samples from serial blood draws (n=18) from patients with a smoking history (n=5) with both targeted and whole-genome sequencing available.<br><br>For the liver cancer cohort, samples were prospectively collected from patients presenting to Johns Hopkins Hospital as part of the HCC biomarker registry at the Johns Hopkins University School of Medicine. Patients included the the study were determined to have either liver cancer (n=48) or cirrhosis of the liver (n=14).
Ethics oversight	The Ethics oversight for the PCAWG protocol was undertaken by the TCGA Program Office and the Ethics and Governance Committee of the ICGC. Each individual ICGC and TCGA project that contributed data to PCAWG had their own local arrangements for ethics oversight and regulatory alignment.<br><br>The LUCAS study was performed according to the declaration of Helsinki and approved by the Danish Regional Ethics Committee (H-2-2011-147) and the Danish Data Protection Agency (j.nr. 2012-58-0004; HEH 750.24.56 and HGH-2018-017; I-Suite nr. 6215).<br><br>All patients in the AHN/DECAMP validation cohort provided written informed consent to participate in these collections and the studies were performed according to the Declaration of Helsinki.<br><br>For the lung cancer monitoring cohort, patient enrollment and genomic studies were conducted in accordance with the Declaration of Helsinki, were approved by the Institutional Review Board and patients provided written informed consent for sample acquisition for research purposes.<br><br>Samples from the liver cancer cohort were collected under a protocol approved by the Johns Hopkins Institutional Review Board. Patients provided written informed consent to participate in these collections and the studies were performed according to the Declaration of Helsinki.

Note that full information on the approval of the study protocol must also be provided in the manuscript.
